# Variable cellular and radiobiological effects of [^177^Lu]Lu-PSMA-I&T in patient-derived models of prostate cancer

**DOI:** 10.1186/s13046-026-03659-w

**Published:** 2026-02-13

**Authors:** Isabel M. Everard, Michael J. de Veer, Edmond M. Kwan, Natalie L. Lister, Shivakumar Keerthikumar, Andrew Ryan, Dinesh Sivaratnam, Mohammad B. Haskali, Gail P. Risbridger, Laura H. Porter, Renea A. Taylor

**Affiliations:** 1 Department of Anatomy and Developmental Biology, Monash Biomedicine Discovery Institute, Cancer Program, Monash University, Clayton, VIC 3800 Australia; 2 Monash Biomedical Imaging, Monash University, Clayton, VIC 3800 Australia; 3 Eastern Health Clinical School, Monash University, Melbourne, Australia; 4 Cancer Services, Eastern Health, Melbourne, Australia; 5 Sir Peter MacCallum Department of Oncology, The University of Melbourne, Parkville, VIC 3010 Australia; 6 Cancer Research Division, Peter MacCallum Cancer Centre, Melbourne, VIC 3000 Australia; 7 TissuPath, Mount Waverley, Melbourne, VIC Australia; 8 Department of Nuclear Medicine, The Royal Melbourne Hospital, Parkville, VIC Australia; 9 GenesisCare Theranostics, Cabrini Hospital, Malvern, VIC Australia; 10 Cabrini Institute, Cabrini Health, Malvern, VIC 3144 Australia; 11 Department of Radiopharmaceutical Sciences, Cancer Imaging, Peter MacCallum Cancer Centre, Melbourne, VIC 3000 Australia; 12 Department of Physiology, Monash Biomedicine Discovery Institute, Cancer Program, Monash University, Clayton, VIC 3800 Australia

**Keywords:** Prostate cancer, Radioligand therapy, PSMA, Tumor microenvironment, Preclinical studies, Patient-derived xenografts, Theranostics, Targeted radiotherapy, Lu-PSMA, Lutetium-PSMA, Tumor heterogeneity, Treatment resistance

## Abstract

**Background:**

[^177^Lu]Lu-PSMA radioligand therapy targets metastatic castration-resistant prostate cancer by delivering radiation to cells expressing prostate-specific membrane antigen (PSMA). While some patients show remarkable responses, up to 50% show little to no benefit, and disease progression inevitably occurs. Eligibility is mainly based on PSMA avidity by PET imaging, yet responses remain highly variable, highlighting a disconnect between PSMA expression and clinical efficacy. This study used PSMA-positive patient-derived xenografts (PDXs) from the Melbourne Urological Research Alliance (MURAL) to explore mechanisms of response and resistance to [^177^Lu]Lu-PSMA treatment.

**Methods:**

PDXs with variable PSMA expression were treated with [^177^Lu]Lu-PSMA-I&T and assessed for radioligand uptake and DNA damage. Histological, genomic and transcriptomic analyses aimed to identify features of radiosensitivity and resistance at a tissue and cellular level.

**Results:**

PDXs recapitulated clinical PSMA histology and exhibit variable responses to [^177^Lu]Lu-PSMA, mirroring the heterogeneity observed in the clinic. Responses ranged from sustained, transient or delayed tumor reduction to treatment resistant, including a remarkable responder with complete tumor regression for up to 20 weeks post treatment. The magnitude and persistence of DNA damage and tumor cell death varied between PDXs, demonstrating divergent radiosensitivity due to intrinsic tumor characteristics. These characteristics included PSMA receptor density, extent of DNA damage, genomic aberrations and baseline transcriptomic signatures.

**Conclusions:**

These findings in PDX tumors emphasize the complexity of predicting [^177^Lu]Lu-PSMA response, beyond what cell line models can capture. Comparable radiation doses with variable regression underscores that radiosensitivity is multifactorial across diverse tumors, supporting the need for integrated multimodal approaches for patient stratification. Integrating baseline genomic and transcriptomic profiles may reveal determinants of [^177^Lu]Lu-PSMA sensitivity to improve patient selection and identify novel combination therapies.

**Supplementary Information:**

The online version contains supplementary material available at 10.1186/s13046-026-03659-w.

## Highlights

- Patient-derived prostate cancer models capture PSMA heterogeneity, providing a robust platform for preclinical theranostic studies.
- Tumor growth response to [^177^Lu]Lu-PSMA is not reliably predicted by PSMA expression level or radioligand uptake.
- Divergent DNA damage response and cell death occurs following [^177^Lu]Lu-PSMA treatment due to intrinsic tumor characteristics.
- Baseline tumor transcriptomic profiles may serve as predictors of response to [^177^Lu]Lu-PSMA therapy.

**Supplementary Information:**

The online version contains supplementary material available at 10.1186/s13046-026-03659-w.

## Background

Recent advancements in the field of nuclear medicine have introduced promising theranostic agents for advanced prostate cancer. [^177^Lu]Lu-PSMA radioligand therapy, which targets prostate-specific membrane antigen (PSMA), has demonstrated significant clinical potential in a subset of patients. However, prostate cancer presents a complex and heterogeneous disease landscape in which the depth and durability of response to [^177^Lu]Lu-PSMA remain poorly understood [1]. A comprehensive understanding of the mechanisms of action and acquired radioresistance to [^177^Lu]Lu-PSMA remain vital for optimising patient treatment strategies.

PSMA expression, a key determinant of [^177^Lu]Lu-PSMA, is upregulated in ~ 65% of advanced prostate tumors [2–4]. Preclinical studies have proposed a direct correlation between PSMA expression and [^177^Lu]Lu-PSMA response in both cell and animal models [5–7]. However, PSMA expression demonstrates marked heterogeneity, both within and between tumors, that is not captured in cell lines [2, 8, 9]. Clinical trials that recruit patients based on high average PSMA expression on PET imaging have demonstrated improved outcomes when PSMA levels are elevated [10, 11]. However, not all patients predicted to benefit from [^177^Lu]Lu-PSMA therapy based on high PSMA have favorable responses, revealing a disconnect between PSMA expression and therapeutic efficacy. This is confirmed by meta-analyses, which demonstrate that the overall biochemical response to [^177^Lu]Lu-PSMA is 46–49%, despite strict patient recruitment criteria [12, 13]. Hence, it is evident that additional underlying mechanisms beyond PSMA expression influence tumor responses to [^177^Lu]Lu-PSMA therapy.

Several clinical predictors of response to [^177^Lu]Lu-PSMA have emerged, such as health status, prior treatments, tumor burden, and metastatic niche [14–17]. However, attempts to reliably predict treatment outcomes based on clinical characteristics are inconsistent across diverse patient cohorts, highlighting the complexity of identifying robust biomarkers for response. Clinical studies focusing on genomic features of [^177^Lu]Lu-PSMA responsive tumors identified a limited number of genomic mutations that were associated with radioresistance, suggesting that additional biological features of tumors play a role in treatment response [18–22]. As such, a number of intrinsic tumor mechanisms have been proposed to underlie treatment failure, including heterogeneous PSMA expression, inadequate or uneven radiation distribution within structurally complex tumors and inherent tumor radioresistance [23, 24]. The diverse range of inherent radiobiological factors remains challenging to characterise and represents a critical area for further investigation. Preclinical studies using heterogeneous patient tumors can effectively elucidate mechanisms of response and resistance, with the potential to improve patient selection and therapeutic outcomes.

In this study, we used serially transplantable patient-derived xenograft (PDX) models of prostate cancer to investigate factors influencing response to [^177^Lu]Lu-PSMA. These models preserve the PSMA expression and cellular heterogeneity, enabling mechanistic interrogation of treatment response and resistance. By integrating preclinical modelling with cancer biology, uropathology and nuclear medicine, we examined genetic and non-genetic determinants of response, including tumor microenvironmental features, PSMA expression heterogeneity and radioligand update, and molecular profiles that may contribute to variable clinical outcomes. This approach was designed to improve understanding of the biological factors underlying response and resistance to PSMA-targeted radioligand therapy.

## Methods

### Patient-derived xenografts

Serially transplantable PDXs were established, maintained and characterized by the Monash Urological Research Alliance (MURAL), as previously described [25–27]. Original specimens of localized prostate cancer tissue were collected from patients undergoing radical prostatectomy or transurethral resection of the prostate, and metastatic prostate cancer tissue was obtained from biopsy, palliative surgery or rapid autopsy through the CASCADE program [28]. Informed, written consent was obtained from patients prior to tissue collection according to human ethics approval from the Cabrini Institute (RES-20-0000-107 C; 03-14-04-08), Monash University (1636; 7996) and Peter MacCallum Cancer Centre (11/102). Established PDXs were either grown in intact 6-8-week-old male non-obese diabetic severe-combined immune-deficient (NSG) mice (RRID: IMSR_JAX:005557) with a 5 mm testosterone pellet implanted subcutaneously to supplement host testosterone levels, or in castrated host mice to mimic androgen deprivation. All animal care and procedures for the establishment and maintenance of PDXs were performed in accordance with Monash University animal ethics approvals (28911; 41088). Routine validation of PDX authenticity included DNA and RNA sequencing, STR profiling and pathological assessment, including biomarker expression for androgen receptor (AR), PSMA and the neuroendocrine markers CD56, synaptophysin and chromogranin A, as previously described [25, 27, 29].

### Animals

All treatment studies were approved by Monash Animal Ethics Committee (31041; 37507). Male NSG mice were bred and housed under controlled temperature (22 °C) and lighting (12:12 h light-dark cycle) and were fed chow diet *ad libitum*. At 6–8 weeks old, intact or castrated mice (dependant on the PDX) were subcutaneously grafted with PDX tissue. Mice were monitored until their tumor volume reached 50 mm^3^ before being transferred to the radiation facility.

### Radioligands and peptide

[^177^Lu]Lu-PSMA-I&T was manufactured and supplied by the Department of Radiopharmaceutical Sciences at the Peter MacCallum Cancer Centre. Carrier-added [^177^Lu]Lutetium chloride (Isotopia Molecular Imaging Ltd) was used to produce [^177^Lu]Lu-PSMA-I&T, with a specific activity of approximately 110 MBq/µg.

### 18 F-PSMA PET imaging

Baseline scans of PSMA expression before treatment were obtained. DCFPyL18F-PSMA was purchased from Cyclotek Australia. For each PDX type, 3 mice were intravenously injected with 10 MBq (± 2 MBq) F18-PSMA, allowed to uptake for one hour, and then imaged for 20 min with positron-emission tomography (PET) using a Mediso Nanoscan PET-CT small animal scanner (Mediso, Budapest, Hungary). PET data was reconstructed using the Teratomo3D mode with 4 iterations and 6 subsets at a voxel size of 0.4 mm. CT used a ZigZag protocol with 360 projections at 50 kVp at 0.67 mAmp and 170 ms exposure time. Reconstruction used a Feldkamp filtered back projection algorithm and a voxel size of 0.125 mm. Images were analysed using PMOD (PMOD LLC, Zurich, Switzerland). SUVmean was calculated from a PET-defined VOI using a relative threshold of 30% of SUVmax. A 30% SUVmax threshold was selected to balance exclusion of background noise while capturing heterogeneous PSMA uptake within tumors, which was particularly relevant for small-volume PDX lesions. CT imaging was used to assist tumor localisation.

### [^177^Lu]Lu-PSMA administration

When tumors reached a minimum volume of 100 mm^3^, mice received a single intravenous injection of 60 MBq ± 10 MBq (total activity) [^177^Lu]Lu-PSMA-I&T (specific activity: 20 MBq/µg; Peter MacCallum Cancer Centre). Control mice were intravenously injected with an equivalent volume of 0.9% sodium chloride. At harvest, PDX tissue was resected and pieces were stored in cryopreservation media (10% DMSO in FBS + 2% ROCK inhibitor) and stored at -80 °C until further processing.

### Flow cytometric analysis of tumor composition

Fresh frozen PDX tissue was thawed and tumor pieces digested in RPMI-1640, containing 0.65 U/mL Liberase TM (Roche) and 0.2 mg/ml DNase I enzymes (Roche), for 1 h at 37 °C. Red blood cell lysis was performed with Red cell Lysis buffer (Sigma) for 1 min and washed with RPMI. Cells were counted and 1-2 × 10^5^ cells were stained for 20 min at 4 °C with relevant antibody concentrations (Supp. Table 1) diluted in FACS buffer (PBS containing 10% FBS and 1 mM EDTA). Cells were washed with FACS buffer and centrifuged at 200 g for 5 minutes to pellet cells. Cells were resuspended in 200uL FACS buffer and analysed on the BD^®^ LSR II Flow Cytometer (BD, Biosciences, NJ, U.S.A). Dead cells were labelled with propidium iodine 30 s prior to sample acquisition. Single colour compensation controls were used for each panel, with unstained voltage controls and isotype controls for each experiment. Data analysis was performed using FlowJo (v10.10).

### Flow cytometric analysis of PSMA receptor density

Frozen tissues were digested and prepared as above. A PE-Quantbrite kit (BD Biosciences # 340495) was used as per manufacturer protocol to quantify the number of PE-PSMA antibodies bound to surface receptors (Supp. Figure 1A, B).

### In vivo PDX radiation uptake

For each PDX type, a minimum of three mice treated with [^177^Lu]Lu-PSMA-I&T were dedicated to a 24-hour harvest timepoint for radiation uptake studies. Tumors were resected, weighed and activity was measured by ex vivo gamma-counting (LabLogic Wiper, Hidex AMG). Data were decay corrected for the time of [^177^Lu]Lu-PSMA-I&T injections and expressed as the percent injected activity per gram of tissue (%IA/g).

### Immunohistochemistry

Tumor tissue was stained for PSMA, yH2Ax, HIF1α and cleaved caspase 3 by immunohistochemistry on formalin fixed tissue (antibody information can be found in Supp. Table 1). Samples were fixed in 10% neutral-buffered formalin until radiation had decayed (~ 65 days), before being fixed in paraffin and sectioned. Tissue sections were stained on the Dako Autostainer Link 48 (High pH Link visualization system reagents) with manual dewaxing in Tris-base solution. Briefly, 5 μm sections were dewaxed and rehydrated, antigen retrieval was performed at 100 °C for 20 min, peroxidase blocking for 10 min, antibody or negative control reagent for 30 min, EnVision Flex HRP polymer for 30 min, DAB for 5 min and finally sections were counterstained using Mayer’s Hematoxylin. Stained sections were fixed and scanned digitally at 20x magnification (Aperio ScanScope AT Turbo slide scanner; performed by Monash Histology Platform).

PSMA localisation was reviewed by a uropathologist, and PSMA expression was manually scored for intensity and percent of total cells using Aperio ImageScope Software (Leica Biosystems). The Shannon index was used to measure heterogeneity of PSMA staining, with three distinct “species” defined as the percent of cells with a staining intensity of + 1, +2 and + 3 respectively. A high value indicates increasing diversity within the sample, representing the probability that two randomly sampled individual cells belong to different species [30–32]. yH2Ax, HIF1α and cleaved caspase 3 were scored using the nuclear v9 algorithm on ImageScope Software for nuclear staining intensity and percent positive cells.

### Measuring tumor growth

Tumor volume was determined twice weekly by calliper measurement of length x width x height x 0.52, as previously described [33]. Mice were humanely euthanised if tumors reached a maximum tumor size of 1000 mm^3^, according to Monash University animal ethics approvals, or at endpoint post-treatment (24 h, 14 days or 10–20 weeks) if tumor volume was < 1000 mm^3^. Tumor tissue was collected for RNA sequencing in RNALater (Invitrogen AM7021), fixed in 10% neutral-buffered formalin for immunohistochemistry analysis, or frozen in cryopreservation media for flow cytometric analysis.

Based on the growth of PDXs over 2–20 weeks, we used pre-defined thresholds for good responders (< 100% of day 0 tumor volume), partial responders (> 100% of day 0 tumor volume, but significantly smaller than average of control group), and non-responders (> 100% of day 0 tumor volume and not significantly different from the average of control group). To facilitate clinically meaningful comparisons of responses in PDX models, we applied modified RECIST criteria, as defined by Meric-Bernstam et al. (2024), to classify individual tumors within each experiment as having a complete response, partial response, stable disease, or progressive disease.

In the long-term experiments, tumor volume was monitored for up to 20 weeks. For these analyses, a ‘sustained response’ was defined as the absence of tumor regrowth before the maximum endpoint, while a ‘transient response’ referred to tumors that initially regressed but eventually grew to a size larger than their day 0 volume at the maximum endpoint. A ‘delayed response’ was characterized by no significant change in tumor volume from day 0 for at least one-week post-treatment, followed by a significant reduction in volume compared to the average of control tumors.

### RNA isolation and bulk sequencing

Total RNA from tumor tissue was isolated using the RNeasy Mini Kit (Qiagen, #74104) according to the manufacturer’s instructions. Total RNA was quantified using a Nanodrop ND-2000 spectrophotometer. Bulk RNA sequencing was performed at Monash Health Translational Precinct, using a custom in-house multiplex method similar to that previously described [34, 35]. Briefly, samples were given a unique i7 index (together with UMI) during individual pA priming and first strand synthesis, which also adds a template switch sequence to the 5’-end. Samples were then pooled into sets and amplified using P7 and an oligo which binds the template switch sequence. Final library construction is completed by tagmentation and addition of unique P5 (with i5 index) by PCR. Sequencing was performed on an Illumina NSQ2k run with 111nt SR (cDNA). An 18nt i7 read contains the 8nt index and 10nt UMI. Samples were parsed using the i7 and i5 indexes.

### RNA and DNA sequencing and gene set enrichment analysis

The quality of the sequenced reads from the PDX samples was initially assessed using FastQC v0.11.6. The reads were then aligned to the reference human genome (hg38) and mouse genome (mm39) with the STAR v2.7.5b aligner. Mouse-specific reads were further filtered using XenofilteR v1.6 and quantified with HTSeq v0.11.2. DESeq2 v1.36.0 [36] was employed to process the count matrix, followed by variance stabilizing transformation (VST). To correct for batch effects in the transformed data, the removeBatchEffect function from the limma v3.52.1 package [37] was used. For gene set enrichment analysis (GSEA), differentially expressed genes (DEGs) were pre-ranked by log2 fold change and tested for pathway enrichments using the gene-based fgsea v1.22.0 R package. The GSEA results are provided as Normalized Enrichment Scores (NES), and this was used to present box plots for post-treatment transcriptomic data. Single-sample GSEA of the PDX samples was conducted using the GSVA R package [38] with the Human Molecular Signatures Database (MSigDB) [39]. The results of the GSEA were visualized in heatmaps and waterfall plots generated with the pheatmap v1.0.12 and ggplot2 functions in R v4.2.0 respectively. The genomic alterations in PDXs, determined by targeted DNA sequencing, was previously published [25].

### Statistical analysis

All data was graphed using Prism (GraphPad, v9 or v10). Linear mixed model analyses were conducted using SPSS Statistics (Version 27; IBM). All other statistical analyses were performed in Prism v9 software (GraphPad). A p-value ≤ 0.05 was considered statically significant. Data analyses were conducted using unpaired Student’s t-test to compare two data sets or using one-way/two-way ANOVA when analysing multiple sets of data. Data were presented as mean ± standard error of the mean (SEM).

## Results

### Variable responses to [^177^Lu]Lu-PSMA treatment in PDXs with high PSMA expression

**Fig. 1:**
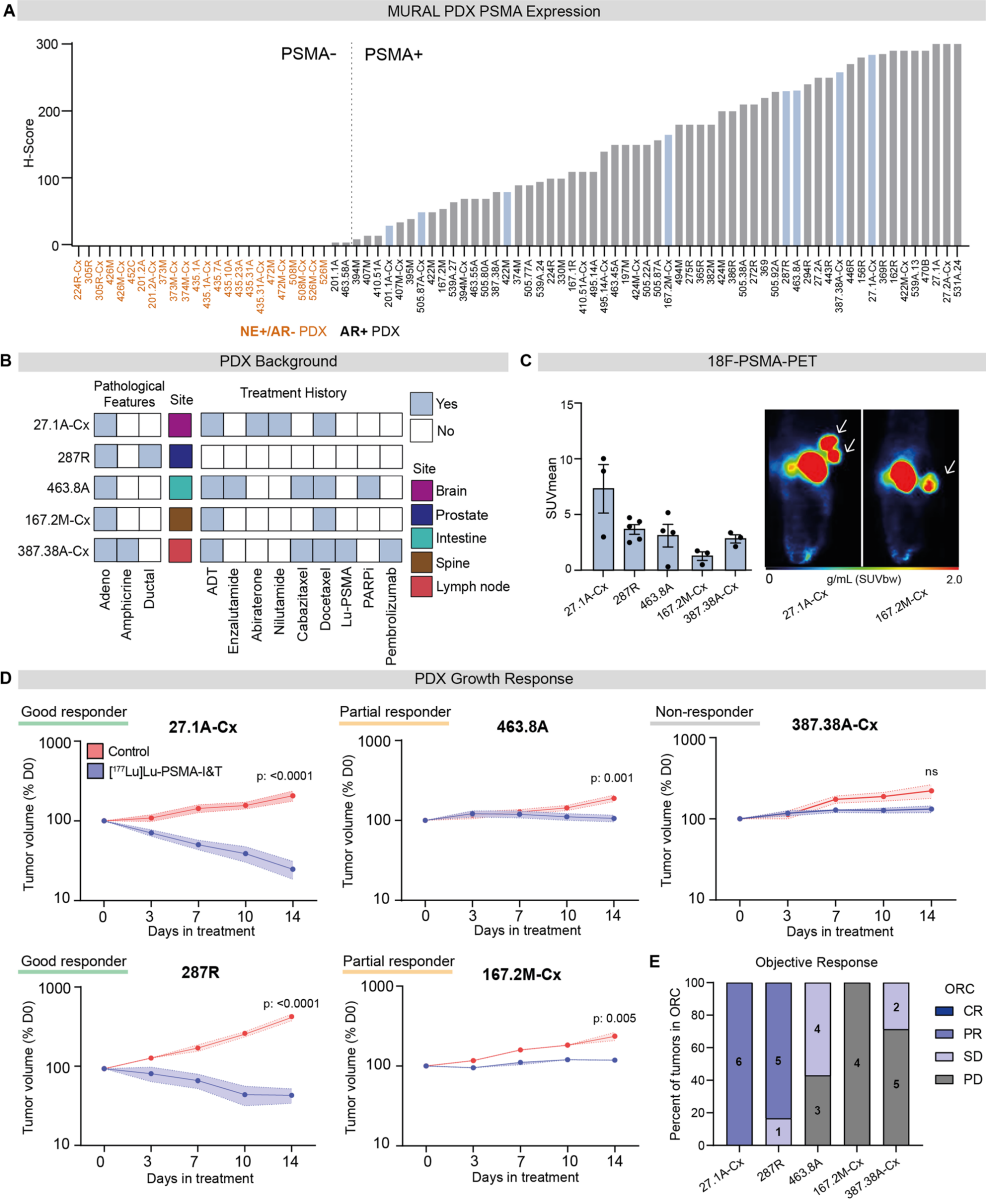
PSMA expression and tumor growth response to [^177^Lu]Lu-PSMA administration in PDXs. **A** PSMA expression in PDXs from the MURAL collection (Risbridger et al. 2021). Dotted line represents the cut off for PSMA positivity (H-score ≥ 10). Blue bars represent PDXs chosen for further analysis. Orange text represents PSMA-negative neuroendocrine tumors. **B** Treatment history and pathology of five PSMA-high tumors from the MURAL PDX collection. **C** PSMA expression by F18-PSMA-PET imaging, expressed as the SUVmean of each tumor (n = 3–5 tumors/PDX), and example PET images of the highest and lowest PSMA-expressing PDXs. White arrows indicate tumors. **D** Tumor volume (mean ± SEM) following intravenous injection of 60 MBq (± 8 MBq) [^177^Lu]Lu-PSMA-I&T or control (saline) on day 0. Sample sizes: 27.1A-Cx n = 6/7, 287R n = 6, 463.8A n = 6/7, 167.2M-Cx n = 4, 387.38A-Cx n = 6/7. P values from unpaired T-test of day 14 volume change. **E** Objective response criteria (ORC) of each tumor in each PDX model, numbers on bars represent numbers of tumors in each group. ADT; androgen deprivation therapy. AR; androgen receptor. CR; complete response. NE; neuroendocrine markers. PARPi; poly (ADP-ribose) polymerase inhibitor. PD; progressive disease. PR; partial response. SD; stable disease

To identify suitable PDX models for [^177^Lu]Lu-PSMA treatment, PSMA expression was initially assessed across the MURAL PDX collection using immunohistochemistry. Consistent with PSMA expression in patient tumors, 67% (56/83) of PDXs were PSMA-positive, ranging from H-Score 10 to 300 (Fig. 1A). All PSMA-positive tumors were also positive for androgen receptor expression (Fig. 1A). In contrast, the majority of PSMA-negative PDXs (H score < 10) were androgen receptor negative and expressed neuroendocrine markers, except for two PDXs that were androgen receptor positive (Fig. 1A).

Eight PDXs with diverse PSMA expression were selected from the MURAL cohort for [^177^Lu]Lu-PSMA treatment (Fig. 1A; Supp. Figure 2A). This included five PDXs with high PSMA expression (PDX-27.1A-Cx, PDX-287R, PDX-463.8A, PDX-167.2M-Cx and PDX-387.38A-Cx), as determined by immunohistochemistry (H-score > 120), as well as three PDXs (PDX-201.1A-Cx, PDX-505.87A-Cx and PDX-422M) with low PSMA expression (H score < 120; Fig. 1A; Supp. Figure 2A). The five PSMA-high PDXs were derived from both localized and metastatic prostate tumors, including brain, intestine, spine, and lymph node metastases, and were from patients with diverse treatment histories (Fig. 1B). All five PDXs are AR-positive adenocarcinomas; however, PDX-287R exhibits ductal architecture, while PDX-387A.8-Cx shows an amphicrine phenotype, co-expressing AR and NE markers (Fig. 1B). Notably, PDX-387A.8-Cx was derived from a patient who had received [^177^Lu]Lu-PSMA therapy prior to tissue collection (Fig. 1B, Supp. Figure 2B). The patient received [^177^Lu]Lu-PSMA late in disease progression after multiple lines of therapy, including androgen deprivation therapy, docetaxel and cabazitaxel (Supp. Figure 2B). Following an initial response to [^177^Lu]Lu-PSMA, the patient progressed with extensive bone disease.

In all PSMA-high PDX models, PSMA expression was confirmed using 18 F-PSMA-PET imaging, with the highest mean standardized uptake value (SUV_mean_) observed in PDX-27.1A-Cx (Fig. 1C; Supp. Figure 2C). PSMA-high PDXs were treated with a single 60 MBq intravenous injection of [^177^Lu]Lu-PSMA. Two PDXs (PDX-27.1A-Cx and PDX-287R) displayed significant reductions in tumor volume after treatment, decreasing to ~ 25% and ~ 46% of starting volume, respectively (Fig. 1D). These tumors were classified as good responders based on a modified RECIST criteria, defined by regression to less than 100% of the baseline tumor volume and a significant reduction in tumor size compared to vehicle-treated controls. Two PDXs (PDX-463.8A and PDX-167.2M-Cx) demonstrated partial responses to treatment with [^177^Lu]Lu-PSMA as both tumors were significantly smaller than vehicle controls at 14 days post-treatment, but did not regress by more than 100% of their baseline volume (Fig. 1D). In contrast, PDX-387.38A-Cx, characterized by an amphicrine phenotype and derived from a patient previously treated with [^177^Lu]Lu-PSMA, was classified as a non-responder, showing no reduction in tumor growth compared to vehicle-treated controls (Fig. 1D). In addition to tumor volume, we assessed PSMA expression by immunohistochemistry in pre- and post-treatment samples and found that PSMA levels in PDXs remained unchanged between control and treatment groups two weeks after treatment (Supp. Figure 2D).

To further compare responses in PSMA-high PDX models with clinically meaningful measurements, an additional modified RECIST criteria, defined by Meric-Bernstram et al. (2024), was used to categorise the growth of individual tumors from each PDX type [40]. These objective response criteria (ORC) mimic radiographic assessments used in patients but are adapted for preclinical settings, where measurements focus on single tumors rather than multiple metastases. Using these criteria, the good responders (PDX-27.1A-Cx and PDX-287R) had 100% and 83% of individual tumors classified as having a complete response respectively (Fig. 1E). In contrast, the partial and non-responders had individual tumors with stable or progressive disease (Fig. 1E). Therefore, although all PDXs exhibited high PSMA expression, their responses to [^177^Lu]Lu-PSMA varied widely, from complete tumor regression to progressive disease, highlighting that PSMA expression alone was not sufficient to predict treatment outcome.

For comparison, three PDXs with low PSMA expression (H score < 120) were also selected for [^177^Lu]Lu-PSMA treatment (Supp. Figure 2A, 3A, 3B). Two of these tumors demonstrated no response to [^177^Lu]Lu-PSMA treatment during the two-week period compared to control grafts (Supp. Figure 3C). In contrast, PDX-505.87A-Cx showed a modest but significant growth attenuation compared to controls at two weeks post [^177^Lu]Lu-PSMA treatment (Supp. Figure 3C). Although PDX-505.87A-Cx is classified as PSMA-low based on average expression levels, individual tumors and tumor sections displayed spatially distinct regions of intense PSMA staining (Supp. Figure 3B, D), which may explain its partial response to treatment. It is also noteworthy that patient 505 received [^177^Lu]Lu-PSMA therapy prior to tissue collection and PDX establishment, suggesting that partial treatment sensitivity may be retained.

### Tumor composition is altered by [^177^Lu]Lu-PSMA treatment

To examine the histological response to [^177^Lu]Lu-PSMA treatment, we analyzed tumor composition by flow cytometry and immunohistochemistry two weeks post-treatment in the five PDX models with high PSMA expression. This approach allowed us to capture the complexity of the tumor microenvironment and cellular changes in PDX tumors following therapy. Consistent with tumor regression following treatment, the good responders (PDX-27.1A-Cx and PDX-287R) showed significant increases in the proportion of EpCAM^-^/CD45^-^ stromal cells and significant decreases in the proportion of EpCAM^+^ epithelial cells in grafts at two weeks post-treatment (Supp. Figure 4A). This was consistent with histological analysis, which revealed extensive regions of stromal cells (Supp. Figure 4B). Despite PDX-167M-Cx and PDX-463.8A showing a partial response to treatment based on tumor size, the proportion of tumor epithelial cells and stromal cells was not altered for these PDXs in treated vs. vehicle control grafts two weeks post-treatment (Supp. Figure 4A, B). Interestingly, PDX-387.38A-Cx, which showed no change in tumor volume in response to treatment, also had a significant loss of EpCAM^+^ epithelial cells and a significant increase in EpCAM^-^/CD45^-^ stromal cells in treated vs. control grafts, similar to the good responders PDX-27.1A-Cx and PDX-287R (Supp. Figure 4A). This is consistent with histological changes seen in treated grafts, where large necrotic areas were evident (Supp. Figure 4B), suggesting that PDX-387.38A-Cx exhibited a biological response to [^177^Lu]Lu-PSMA treatment, despite no overall change in graft size. The immune cell composition within tumors, which is limited in NSG mice, varied between PDX models but did not show significant changes after treatment in any PDX model (Supp. Figure 4A). Overall, the two good responders (PDX-27.1A-Cx and PDX-287R) had pronounced histological changes that corresponded with a reduction in tumor burden; however, [^177^Lu]Lu-PSMA may also cause compositional changes within some tumors that is not reflected in overall tumor volume.

### Variable long-term responses to [^177^Lu]Lu-PSMA treatment in PDXs with high PSMA expression

**Fig. 2:**
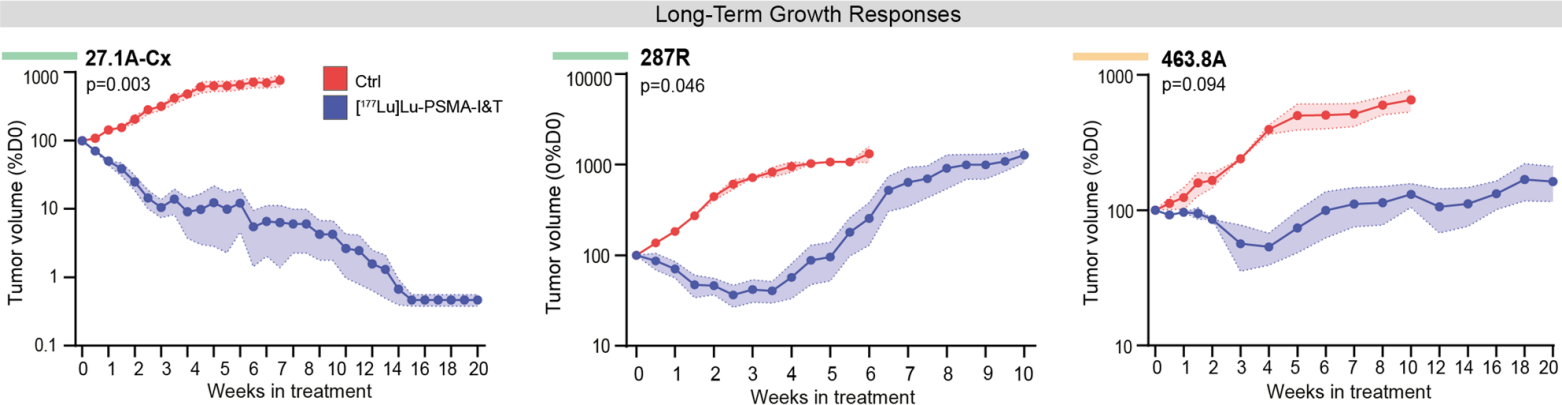
Long-term responses to [^177^Lu]Lu-PSMA. Tumor volume (mean ± SEM) following intravenous injection of 60 MBq (± 8MBq) [^177^Lu]Lu-PSMA-I&T or control (saline) on day 0, measured over a maximum of 20 weeks (*n* = 3 mice per group). Overall P values from mixed model analysis. Green and orange bars indicate good and partial responses respectively, determined in Fig. 1

Three PDXs were selected for long-term growth analysis, including the two good responders (PDX-27.1A-Cx and PDX-287R) and one partial responder (PDX-463.8A). Following a single 60 MBq administration of [^177^Lu]Lu-PSMA, PDX-27.1A-Cx demonstrated a substantial and persistent reduction in tumor volume, diminishing to less than 1% of the initial volume over the 20-week monitoring period with no subsequent tumor regrowth (Fig. 2). By the 20-week endpoint, there was complete tumor regression with no analysable tumor tissue (Fig. 2). In contrast, the other good responder (PDX-287R), a treatment naïve primary prostate cancer with ductal pathology, exhibited a significant reduction in tumor volume compared to vehicle-treated control over a 2- to 5-week period, but subsequently resumed active growth (Fig. 2). By 10 weeks post treatment, PDX-287R tumors had regrown and were collected for histological analysis when they reached the maximum ethical limit of 1000 mm^3^ (Fig. 2). At this time point, immunohistochemistry for the epithelial cell marker ck8/18 showed no difference in histology between [^177^Lu]Lu-PSMA and vehicle-treated tumors (Supp. Figure 5). In contrast to PDX-27.1A-Cx and PDX-287R, which exhibited immediate tumor regression following [^177^Lu]Lu-PSMA treatment, the partial responder (PDX-463.8A) showed a delayed response to treatment, with tumor regression not occurring until 2 weeks post-treatment (Fig. 2). This response was transient as tumor growth later resumed at a reduced rate (Fig. 2). However, this reduction and delayed growth was not statistically significant compared to vehicle-treated controls based on mixed-model analysis (Fig. 2). At the experimental endpoint 20 weeks post treatment, PDX-463.8A tumors exhibited large necrotic areas that were not present in vehicle-treated controls (Supp. Figure 5), indicating persistent treatment-induced cellular changes in tumor morphology. The responses observed in these three PDX models underscore the variability in both onset and duration of therapeutic responses to [^177^Lu]Lu-PSMA among PSMA-high tumors. This included divergent long-term responses in the two good responders, with a sustained response in PDX-27.1A-Cx and a transient response in PDX-287R.

### PSMA expression profiles reveal intratumoral complexity in PDX models

**Fig. 3:**
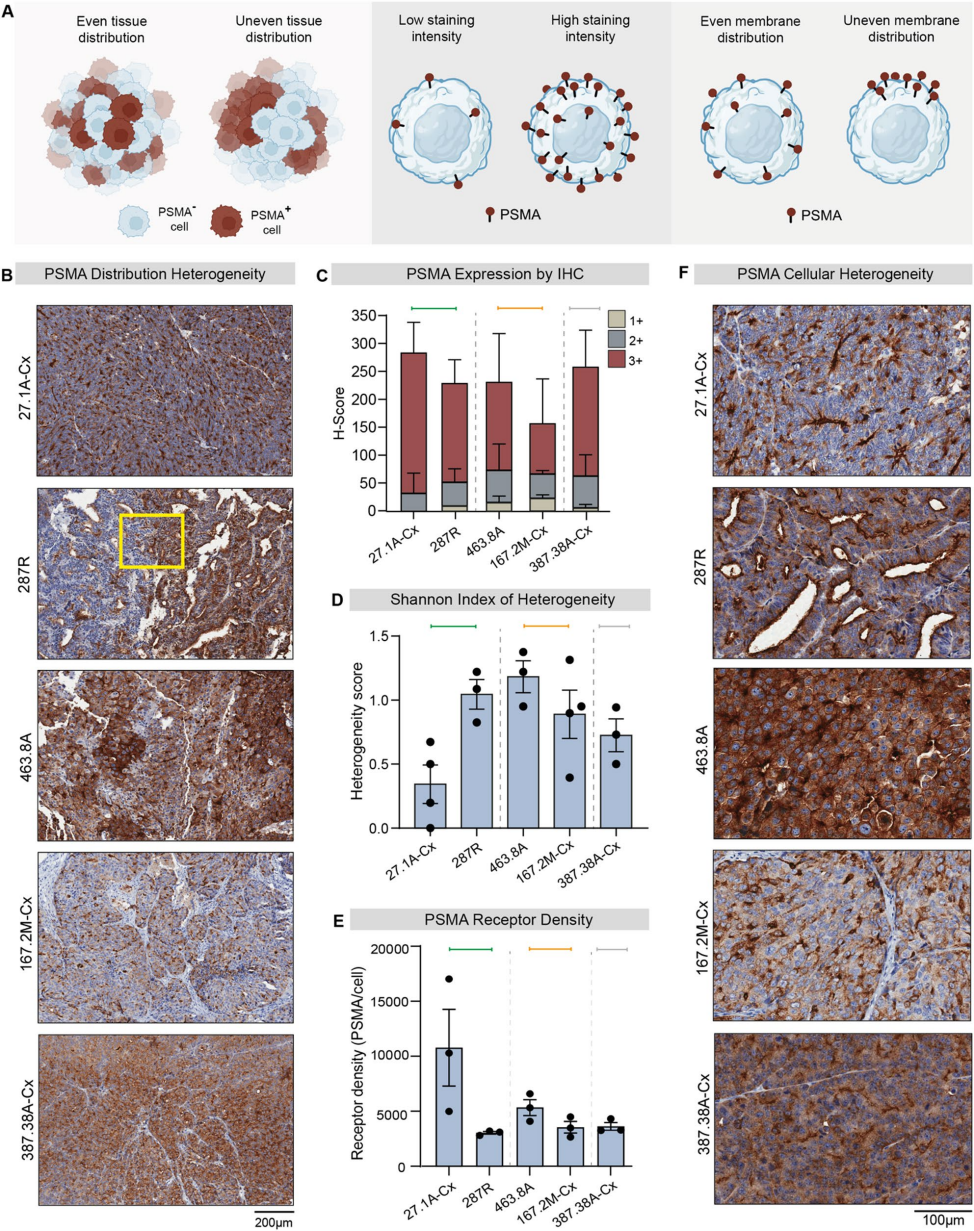
Intratumoral PSMA heterogeneity in prostate cancer PDXs.** A** Schematic showing different levels of PSMA heterogeneity, including tissue distribution (left), staining intensity (middle), and receptor localisation (right). **B **Immunohistochemical (IHC) PSMA staining showing intratumoral heterogeneity. Yellow box indicates PSMA-high next to PSMA-low regions. **C **PSMA expression quantified by H-Score via IHC, calculated based on the number of cells expressing PSMA (as % of the whole tumor) and their staining intensity on a scale from 1 to 3 (*n* = 3–4 PDX per group). **D **Shannon index of tumor heterogeneity calculated on control samples. *n* = 3–4 tumors per PDX. **E **PSMA receptor density measured by cytometric bead assay (*n* = 3 tumors per PDX). **F** IHC PSMA staining in individual representative tumors showing PSMA cellular localisation. Coloured bars indicate growth responses determined in Fig. 1 (green, good responder; orange, partial responder; grey, non-responder)

Although the five PDXs were classified as PSMA-high on average, we hypothesized that intratumoral PSMA heterogeneity might influence responses to [^177^Lu]Lu-PSMA treatment. PSMA expression varies widely at the tumor and cellular levels in intensity, localization, and spatial distribution (Fig. 3A), which is not always detectable by PSMA-PET due to its limited cellular resolution.

Using immunohistochemistry, we first observed that PSMA-high and PSMA-low regions can exist next to each other in the same tumor, as in PDX-287R, compared to other tumors where PSMA-positive cells are dispersed evenly throughout, such as in PDX-27.1A-Cx and PDX-387.38A-Cx (Fig. 3A, B). In addition to distinct PSMA-high and PSMA-low regions in some PDXs, we also observed differences in PSMA staining intensity across the entire tumor for all PDXs (Fig. 3A, C). To quantify staining intensity, we used the immunohistochemistry H-Score system, which grades membrane staining intensity on each cell from low to high (1+, 2+, or 3 + intensity). The sustained good responder (PDX-27.1A-Cx) had the highest proportion of strong staining intensity, comprising almost 100% 3 + cells, while all other PDXs had at least a proportion of cells with low (+ 1) or moderate (+ 2) staining intensity (Fig. 3C). To formally quantify variations in PSMA expression intensity, we used the Shannon heterogeneity index (Fig. 3D) [30–32]. Consistent with the H-score, PDX-27.1A-Cx exhibited the lowest heterogeneity score (Fig. 3D), indicating the most uniform PSMA staining within individual tumors. However, the heterogeneity score did not correlate with response to [^177^Lu]Lu-PSMA treatment in the remaining PDXs; notably, the other good responder (PDX-287R) exhibited a heterogeneity score comparable to those of the partial and non-responders (Fig. 3D). Thus, while uniform PSMA expression within tumors may be associated with response in some contexts, it is not a consistent predictor across models. Importantly, the small number of PDX samples analyzed limits the ability to draw definitive conclusions.

As high cytoplasmic PSMA staining can confound membrane scoring by immunohistochemistry, we also quantified membrane PSMA receptor density using a flow cytometric bead assay. PDX-27.1A-Cx had the highest PSMA receptor density per cell, although this was not significantly different to the other PDXs due to variations between individual tumors (Fig. 3E). Interestingly, PDX-287R had similar receptor density to the partial- and non-responding PDXs (PDX-167.2M-Cx, PDX-463.8A and PDX-387.38A-Cx; Fig. 3E), suggesting that high PSMA receptor density per cell may not always be essential for treatment responsiveness.

In addition to measuring PSMA intensity and density, differences in PSMA cellular localisation were examined between PDXs (Fig. 3A). Interestingly, the good responders (PDX-27.1A-Cx and PDX-287R) demonstrated high PSMA staining on the apical surface of cells adjacent to either open or compressed luminal spaces (Fig. 3F). In comparison, PDX-167.2M-Cx, PDX-463.8A and PDX-387.38A-Cx had homogenous PSMA staining around the membrane and cytoplasm (Fig. 3F).

Collectively, these findings in preclinical models illustrate the multi-level heterogeneity of PSMA expression within individual tumors. While the sustained responder (PDX-27.1A-Cx) showed uniform, high apical expression and PSMA receptor density, PDX-287R responded despite lower receptor density and focal areas of low PSMA expression. Overall, no single PSMA expression profile clearly predicted response to [^177^Lu]Lu-PSMA therapy in this set of PDX models.

### Treatment-induced cell death does not always reflect radioligand uptake in PDXs

**Fig. 4:**
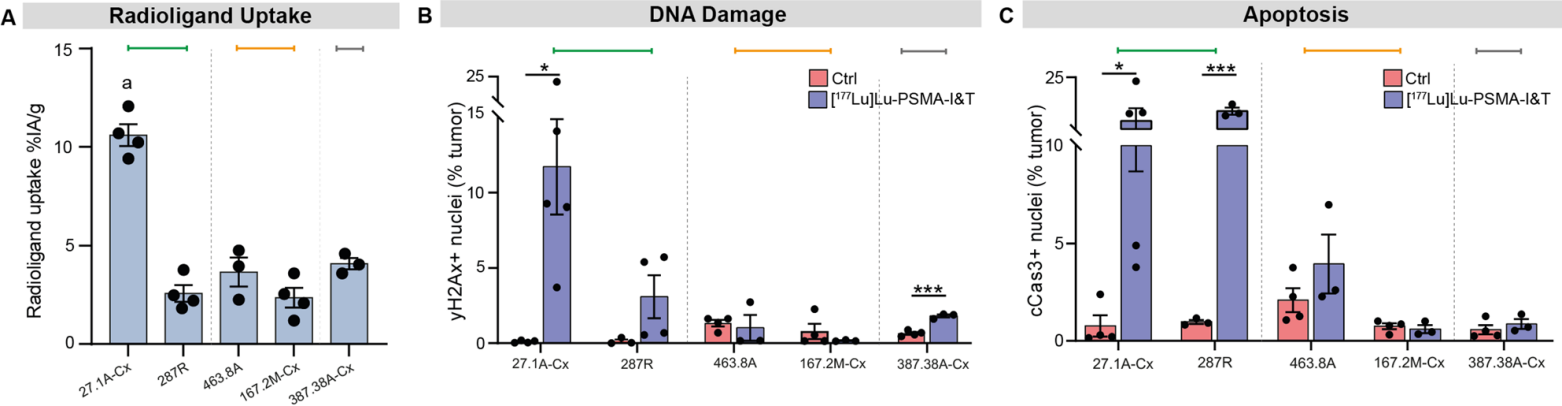
Radiation uptake, DNA damage and cell death after treatment with [^177^Lu]Lu-PSMA.** A **Whole tumor radiation uptake, measured by a gamma counter 24 h after [^177^Lu]Lu-PSMA-I&T administration and expressed as the percent of injected activity per gram of tumor tissue (%IA/g; *n* = 3–4 tumors/PDX). One-way ANOVA with Tukey’s multiple comparisons between PDX-27.1A-Cx compared to all other groups (a = *p* < 0.0001). **B**,** C **Expression of DNA damage repair marker yH2Ax (**B**) and apoptotic marker cleaved caspase 3 (cCasp3; **C**) at 24 h post [^177^Lu]Lu-PSMA, quantified by immunohistochemical staining (*n* = 3–5 tumors/PDX). Unpaired students t-test between control and treated tumors within the same PDX model (*p < 0.05 ***p < 0.001). Coloured bars indicate growth responses determined in Fig. 1 (green, good responder; orange, partial responder; grey, non-responder)

Due to the variations in intratumoral PSMA expression and response to treatment between PDXs, we next sought to assess the uptake of [^177^Lu]Lu-PSMA within the tumors. We measured gamma radiation emitted from resected tumors 24 h after a single 60 MBq injection of [^177^Lu]Lu-PSMA. Tumors with high PSMA expression all exhibited radioligand uptake (Fig. 4A), compared to PSMA-low PDXs (Supp. Figure 6A). PDX-27.1A-Cx, which had a sustained response to treatment, exhibited significantly higher radioligand uptake compared to all other PDXs (Fig. 4A), consistent with its high PSMA expression by PET imaging and immunohistochemistry, and high receptor density by flow cytometry (Figs. 1A and 1C and 3E). In contrast, the other good responder (PDX-287R) had similar radioligand uptake to the partial responders and non-responder (PDX-463.8A-Cx, PDX-167.2M-Cx, and PDX-387.38A-Cx respectively), and significantly lower radiation uptake compared to PDX-27.1A-Cx (Fig. 4A), which is consistent with the lower PSMA receptor density per cell in these PDXs compared to PDX-27.1A-Cx (Fig. 3E).

To investigate why PDX-287R demonstrated greater responsiveness to treatment despite similar levels of radioligand uptake, we examined DNA damage and apoptosis following [^177^Lu]Lu-PSMA therapy. This was assessed by immunohistochemistry using γH2Ax as a marker of DNA double-strand breaks and cleaved caspase-3 (cCas3) as an indicator of apoptosis. As expected from its high radioligand uptake, PDX-27.1A-Cx exhibited significantly increased levels of γH2Ax and cleaved caspase-3 at 24 h post-treatment compared to vehicle-treated controls (Fig. 4B, C). Interestingly, although PDX-287R showed only a modest, non-significant increase in DNA damage at 24 h post-treatment, levels of cleaved cCas3 were significantly elevated compared to controls and comparable to those observed in PDX-27.1A-Cx (Fig. 4B). In contrast, the partial responders (PDX-463.8A and PDX-167.2M-Cx) showed no significant increases in γH2Ax or cCas3 at 24 h post-treatment, despite exhibiting similar whole-tumor radiation uptake to PDX-287R (Fig. 4B, C). These findings suggest that PDX-287R may be more sensitive to radiation-induced DNA damage, leading to greater tumor cell apoptosis and regression compared to other models. However, this treatment sensitivity appeared to be transient, as γH2Ax and cCas3 staining returned to control levels by two weeks post-treatment in PDX-287R (Supp. Figure 6B, C). In contrast, detection of these markers remained significantly higher than controls by two weeks post treatment in PDX-27.1A-Cx (Supp. Figure 6B, C). This persistent sensitivity to a single injection of [^177^Lu]Lu-PSMA treatment may explain the sustained tumor regression seen in PDX-27.1A-Cx compared to the transient response seen in PDX-287R.

Surprisingly, although PDX-387.38A-Cx showed significant epithelial tumor cell loss by flow cytometry and extensive necrosis, it exhibited only a small but significant increase in γH2Ax at 24 h post-treatment (Fig. 4B), with no significant accumulation at two weeks (Supp. Figure 6B). Notably, no significant increases in cCas3 were observed at either 24 h or two weeks post-treatment (Fig. 4C; Supp. Figure 6C). Therefore, despite comparable whole-tumor radiation uptake, the extent of DNA damage and apoptosis varied markedly between PDX models, indicating that these factors alone do not account for radiosensitivity or tumor growth response.

### PDXs undergo variable transcriptomic changes in response to [^177^Lu]Lu-PSMA treatment

**Fig. 5:**
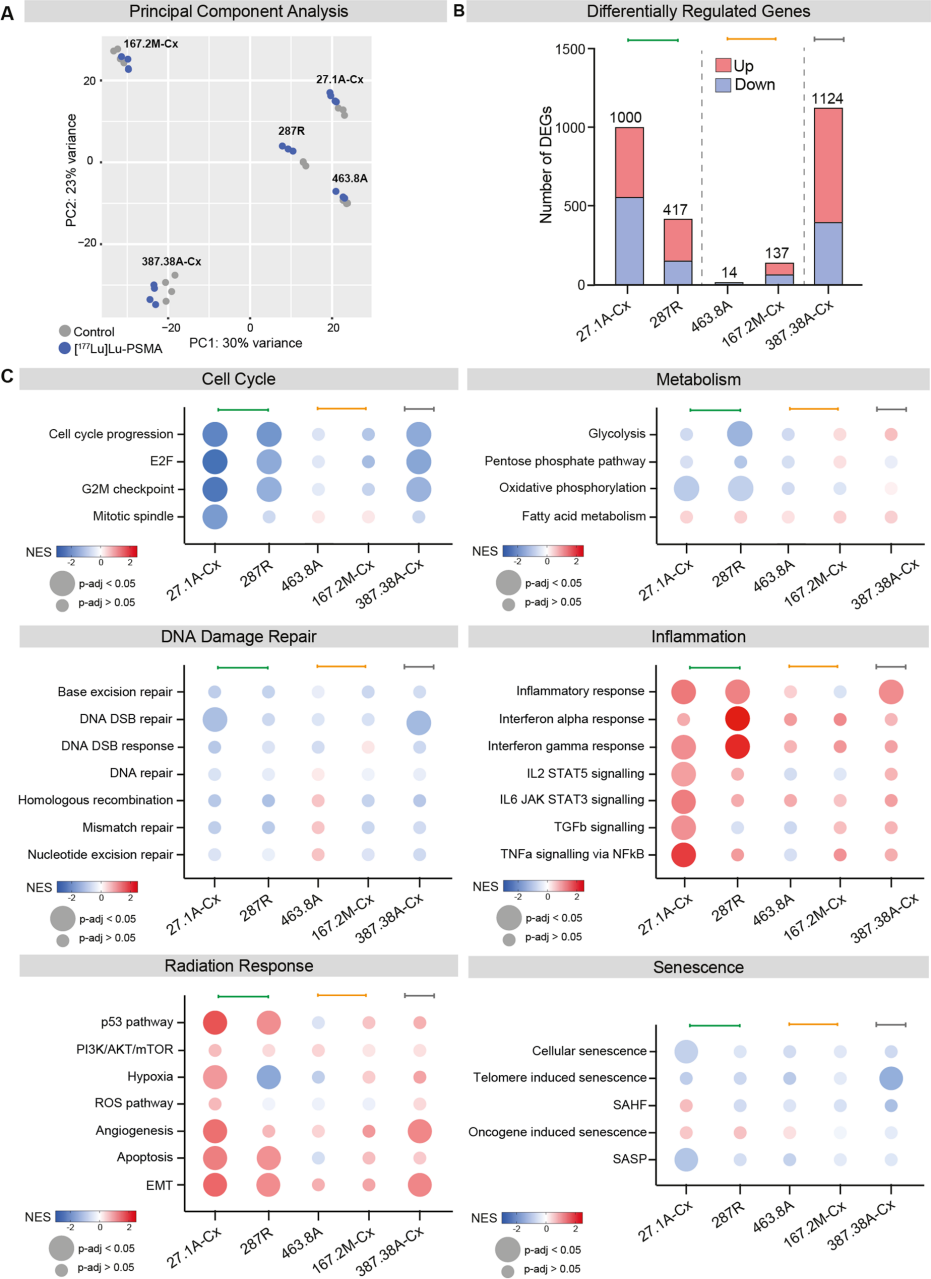
Transcriptomic regulation in PDXs after [^177^Lu]Lu-PSMA treatment. RNAseq samples were collected 14 days after treatment with a single 60 MBq injection of [^177^Lu]Lu-PSMA and compared to control samples for each PDX (n = 3/4 PDX per group). **A** Principal component analysis plot of PDX control and treated samples, highlighting variance between and within PDX. **B** Number of significantly differentially up- and down-regulated genes in each PDX 14 days post-treatment with [^177^Lu]Lu-PSMA. **C** Normalized enrichment scores (NES) grouped by hallmark cancer pathways sharing similar functions. Circle colour shows NES between PDXs and circle size denotes NES that are significantly different between [^177^Lu]Lu-PSMA-treated and vehicle-treated tumors from the same PDX model (adjusted p value < 0.05). Coloured bars indicate growth responses determined in Fig. 1 (green, good responder; orange, partial responder; grey, non-responder). DEG; differentially expressed gene. DSB; double strand break. EMT; epithelial-mesenchymal transition. NES; normalized enrichment score. ROS; reactive oxygen species. SAHF; senescence-associated heterochromatin foci. SASP; senescence-associated secretory phenotype

To determine whether transcriptomic changes were associated with PDX responsiveness to radiotherapy, we analysed the transcriptomes of control and treated PDX samples two weeks after [^177^Lu]Lu-PSMA treatment. Principal component analysis (PCA) revealed distinct transcriptomic profiles between PDX lines (Fig. 5A). Assessing differentially expressed genes (DEGs) with an adjusted p-value (false discovery rate) < 0.05, the two good responders (PDX-27.1A-Cx and PDX-287R) exhibited 1000 and 417 DEGs, respectively, while the partial responders (PDX-463.8A and PDX-167.2M-Cx) showed only 14 and 137 DEGs, respectively (Fig. 5A). Interestingly, PDX-387.38 A-Cx, classified as a non-responder, displayed the highest number of DEGs at 1124, surpassing even the sustained responder PDX-27.1A-Cx (Fig. 5A).

Normalized enrichment scores were compared across PDX models following [^177^Lu]Lu-PSMA treatment to identify alterations in hallmark cancer pathways compared to vehicle-treated tumors. A comprehensive list of hallmark cancer pathways and their significance can be found in Supp Fig. 7. There was a significant downregulation in cell cycle pathways in the good responders (PDX-27.1A-Cx and PDX-287R) and the non-responder (PDX-387.38A) compared to vehicle-treated tumors, but not the partial responders (Fig. 5C). DNA damage repair pathways exhibited similar trends; however, these were predominantly not significant (Fig. 5C). Conversely, good responders demonstrated significant upregulation of pathways associated with radiation response and inflammation, including the p53, interferon, TNFα and TGFβ signalling pathways (Fig. 5C). There was also evidence for microenvironmental adaptation (changes in hypoxia, angiogenesis and epithelial-mesenchymal transition pathways), metabolic changes (significant downregulation of glycolysis and oxidative phosphorylation) and increased cell death (significant upregulation in the apoptotic pathway) in the good responders (Fig. 5C). We also assessed senescence pathways to determine whether an upregulation in senescence may occur in partially-responsive tumors that did not maintain the same growth rate as controls, but did not have a reduction in tumor burden following treatment. However, there was no upregulation in senescence signatures in the partial responders (PDX-463.8A and PDX-167.2M-Cx; Fig. 5C). Collectively, [^177^Lu]Lu-PSMA treatment induced an inflammatory phenotype with microenvironmental changes and apoptosis, together with suppression of cell cycle pathways, in the good responders that was not seen in partially responsive tumors.

Interestingly, the non-responder (PDX-387.38A-Cx) had similar transcriptome alterations in all hallmark cancer pathways as both good responders (Fig. 5C). The high number of DEGs, along with the downregulation of cell cycle pathways and observed histological changes, suggests that PDX-387.38A-Cx is biologically responsive to radioligand therapy despite showing no change in overall tumor volume.

### Baseline genomic and transcriptomic features of PDXs may influence response to [^177^Lu]Lu-PSMA treatment

**Fig. 6:**
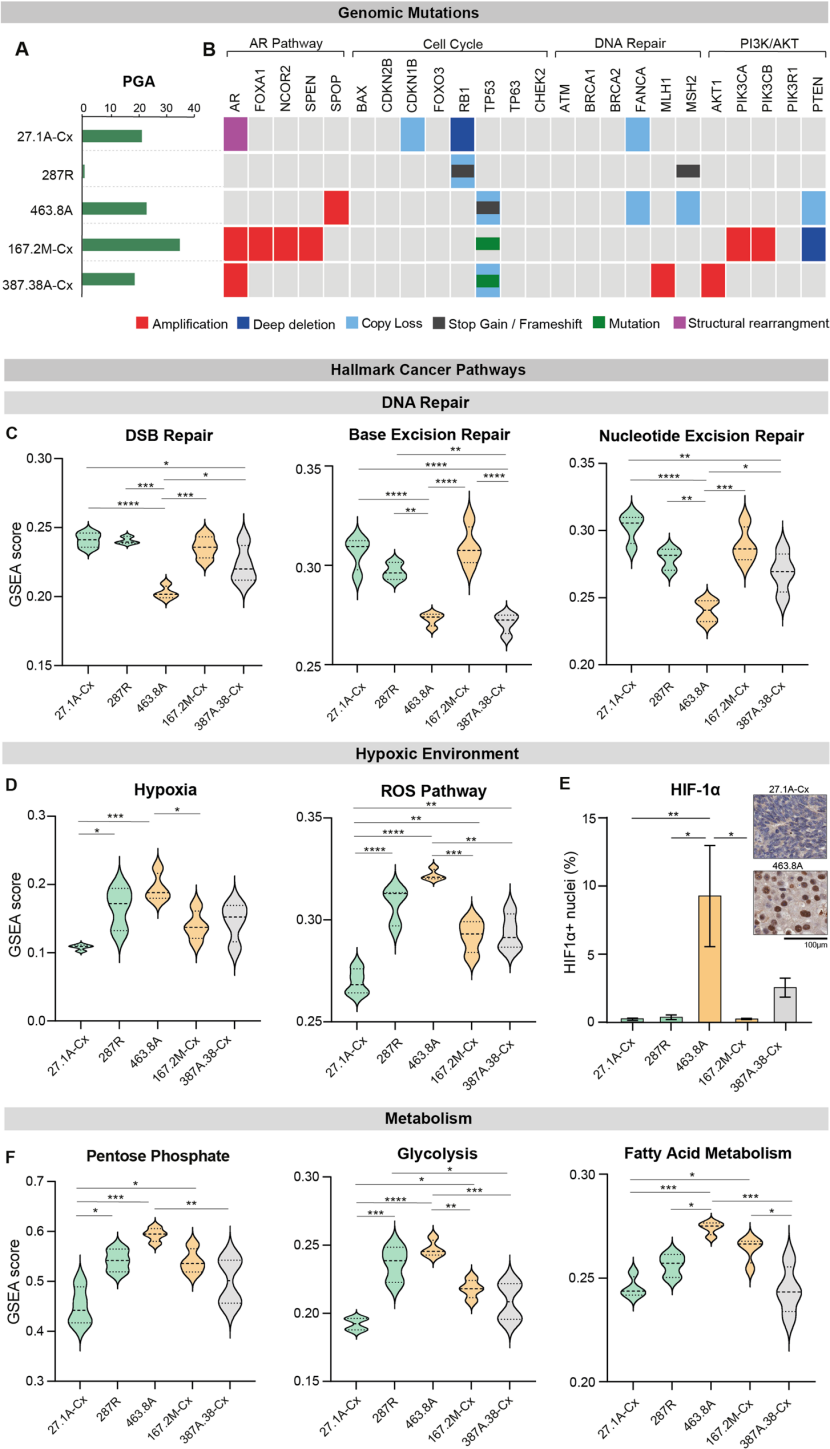
Baseline genomic and transcriptomic features of prostate cancer PDXs. **A** Percent genome alteration across all genes in untreated PDXs. **B** Genomic mutations in a subset of genes related to radiation response in untreated PDXs. **C, D, F** Single-sample gene set enrichment of hallmark cancer pathways related to DNA repair, radiation response and metabolism. **E** Quantification of HIF1a immumohisochemistry in untreated tumors, with representative images from a PDX with low (PDX-27.1A-Cx) and high (PDX-463.8A) HIFa expression respectively (n = 3/4 tumors per PDX). Statistics from one-way ANOVA with Tukey's multiple comparisons test (*p < 0.05, **p < 0.01, ***p < 0.001, ****p < 0.0001). Coloured violins and vars indicate growth responses, as determined in Fig. 1 (green, good responder; orange, partial responder; grey, non-responder). PGA; percent genome alteration. GSEA; gene set enrichment analysis. DSB; double strand break. HIF1a; hypoxia-inducible factor 1a

In addition to treatment-induced transcriptomic changes, we also considered whether baseline characteristics of PDX models could predict responses to [^177^Lu]Lu-PSMA treatment. To evaluate this, we compared genomic and transcriptomic features of untreated tumors. Given that a high mutational burden is often associated with advanced prostate cancer and poorer prognosis, as well as predicting radiotherapy response in other cancers [41, 42], we began by assessing the percentage of genome alterations (PGA) across the PDX models (Fig. 6A). The good transient responder (PDX-287R) exhibited the lowest PGA, consistent with its origin from a treatment-naïve localized prostate tumor. In contrast, the other good responder (PDX-27.1A-Cx) showed a PGA comparable to both the partial responder PDX-463.8A and the non-responder PDX-387.38 A-Cx (Fig. 6A). Notably, the partial responder PDX-167.2M-Cx had the highest PGA among the cohort (Fig. 6A). These results indicate that PGA does not appear to predict response to [^177^Lu]Lu-PSMA treatment in this set of PDX models.

Using targeted DNA sequencing, we next examined baseline genomic features of the PDX models, focusing on copy number variations and high allele frequency mutations in genes commonly altered in prostate cancer and associated with radioresistance [25, 43–45]. The sustained responder (PDX-27.1A-Cx) harboured mutations in *CDKN1B* and *RB1*, both linked to increased radiosensitivity [46, 47], as well as loss of the DNA repair gene *FANCA* (Fig. 6B), which may enhance radiation sensitivity. PDX-287R, which demonstrated a transient response, exhibited biallelic *RB1* inactivation and a frameshift mutation in *MSH2* (Fig. 6B), a DNA repair gene protective against oxidative damage [48], potentially contributing to its treatment sensitivity.

In contrast, PDX-463.8A also had alterations in *FANCA* and *MSH2*, but additionally carried a *TP53* mutation known to confer radiation resistance (Fig. 6B). The other partial responder (PDX-167.2M-Cx) and the non-responder (PDX-387.38A-Cx) both harboured *TP53* mutations and aberrations in the PI3K pathway (Fig. 6B), both of which are implicated in reduced radiosensitivity. *TP53* mutations can lead to gain-of-function phenotypes, impacting DNA repair and reactive oxygen species regulation [49–52]. Notably, none of the PDXs carried *CHEK2* mutations, which have been associated with castrate-resistant progression [53, 54] but also linked to favorable responses to radioligand therapy [55].

In addition to genomic profiling, we assessed baseline transcriptomic features of the PDXs using RNA sequencing and gene set enrichment analysis (GSEA). We initially hypothesized that differences in DNA damage repair pathway activity might underlie treatment sensitivity. However, both good responders (PDX-27.1A-Cx and PDX-287R) exhibited relatively high GSEA scores for DNA repair signatures (Fig. 6C). Conversely, PDX-463.8A, which failed to accumulate DNA damage post-treatment, showed significantly lower scores in single- and double-strand break repair pathways (Fig. 6C). These findings suggest that baseline enhancement of DNA damage repair pathways does not explain the observed resistance to DNA damage in these PDX models.

We next examined baseline gene expression related to hypoxia and reactive oxygen species pathways, given that tumor oxygenation and adaptive responses to low oxygen levels are closely linked to radiation sensitivity [56]. Hypoxic tumors are often more radioresistant due to factors such as reduced production of reactive oxygen species (ROS) and enhanced antioxidant capacity to neutralize ROS, which together diminish radiation-induced DNA damage. Interestingly, gene sets associated with hypoxia and ROS responses were significantly downregulated in the sustained good responder (PDX-27.1A-Cx; Fig. 6D). Thus, the baseline transcriptomic profile of PDX-27.A-Cx may have contributed to its enhanced treatment sensitivity. In contrast, the partial delayed responder (PDX-463.8A), which showed no accumulation of DNA damage following [^177^Lu]Lu-PSMA treatment (Fig. 4B), exhibited significantly higher GSEA scores for hypoxia and ROS pathways (Fig. 6D), indicating a radio-protective hypoxic microenvironment with elevated antioxidant capacity. To validate this at the protein level, we performed immunohistochemical staining for HIF1α, a key regulator of hypoxia that is also upregulated by high ROS levels. Consistent with the transcriptomic data, PDX-463.8A displayed significantly increased nuclear HIF1α staining compared to both good responders (Fig. 6E), supporting the presence of a hypoxic environment that may contribute to its radioresistance.

In response to a hypoxic tumor microenvironment, metabolic reprogramming can further potentiate radiation resistance. The glycolytic, fatty acid, and pentose phosphate pathways have been linked to radioresistance by promoting DNA damage repair [56]. Consistent with the radio-protective environment, PDX-463.8 A had significantly higher GSEA scores for these metabolic pathways compared to the good responder (PDX-27.1A; Fig. 6F). However, the other good responder (PDX-287R) did not have low GSEA scores for hypoxia, ROS and the metabolic pathways, potentially demonstrating different avenues of treatment sensitivity across different tumors, or reflecting the transient treatment response.

Together, these findings highlight that baseline transcriptomic signatures of hypoxia, oxidative stress, and metabolic reprogramming may indicate underlying mechanisms of radiosensitivity and resistance in a subset of tumors.

## Discussion

[^177^Lu]Lu-PSMA therapy is used to treat metastatic castrate-resistant prostate cancer, but patient responses remain highly variable despite strict selection criteria. To investigate the basis of this variability, we examined responses to [^177^Lu]Lu-PSMA therapy in PSMA-positive prostate cancer PDX models, which preserve the complexity and genetic diversity of human tumors in an in vivo context. Primary and acquired resistance to [^177^Lu]Lu-PSMA in patients is partially attributed to clinical factors, including undetectable PSMA-negative lesions, metastatic site and sub-optimal radiation delivery, particularly to micrometastases that fall below the effective path length of β-particles emitted by Lutetium-177 [23, 24]. However, our findings in PSMA-high PDXs, where such confounders are minimised, clearly demonstrate that underlying biological mechanisms also play a key role in therapeutic efficacy. Uniform PSMA expression and high receptor density is likely to contribute to high radioligand uptake and treatment response, given the sustained response seen in PDX-27.1A-Cx. However, radioligand uptake alone did not always correlate with tumor regression, DNA damage, or apoptosis, suggesting that intrinsic tumor features also contribute to radiosensitivity. These features appear to be multifactorial and tumor dependent, with baseline transcriptomic signatures potentially playing a role in influencing treatment efficacy. This highlights a need to further validate the incorporation of molecular biomarkers with PSMA expression and genomic profiles to refine patient stratification and improve outcomes.

Radioligand therapy is based on the principle that targeted internalisation delivers ionising radiation to tumor cells, causing direct damage to macromolecules and inducing DNA damage that activates cell death pathways, including necrosis and apoptosis [57]. In vitro studies using cancer cell lines have demonstrated a near-linear correlation between PSMA expression, radioligand uptake and DNA damage, with higher levels of PSMA expression leading to greater intracellular accumulation and thus more pronounced cytotoxic effects [5–7]. In the clinic, this response is not as simplistic, with PSMA heterogeneity, both within and between lesions, and underlying tumor radiosensitivity impacting treatment response [58]. Several dosimetry studies suggest the importance of tumor absorbed dose, with higher tumor absorbed doses corresponding with prostate-specific antigen response [59, 60]. However, the relationship between PSMA expression, absorbed tumor dose and radiobiological response requires further investigation.

In our studies using PDX models, absorbed radiation appeared to play a key role in response, with the highest radiation uptake seen in the sustained responder, PDX-27.1A-Cx. However, despite similar levels of radioligand uptake across the other PDX models, we observed considerable variability in γH2Ax expression and tumor cell apoptosis, suggesting that in some cases there is a disconnect between radiation uptake and subsequent DNA damage and cell death. For example, PDX-463.8A showed moderate radiation uptake but no detectable DNA damage at 24 h or 14 days post-treatment. In contrast, PDX-287R, with a comparable radiation uptake, exhibited significant cell death despite only moderate DNA damage, suggesting that some tumors have intrinsic radiosensitivity independent of radioligand uptake or damage extent. This suggests that radiation uptake alone does not guarantee therapeutic effect, and that tumor-intrinsic factors, such as DNA repair capacity, chromatin organisation, or microenvironmental context, may significantly modulate radiosensitivity. These observations highlight the limitations of extrapolating cell line data and underscore the importance of investigating the molecular and cellular determinants of radioligand response in more physiologically relevant models.

In this study, we focused on investigating biological determinants of treatment response following a single administration of [^177^Lu]Lu-PSMA. Currently, in the clinic, patients receive multiple cycles of [^177^Lu]Lu-PSMA at six weekly intervals; however, durable responses are rare, and disease progression inevitably occurs. Further studies in these PDX models, for instance in PDX-287R which showed a transient response to a single cycle, are warranted to investigate whether the same intrinsic factors play a role in acquired treatment resistance and explore the emergence of radioresistant tumor features.

We examined genetic alterations as intrinsic factors influencing sensitivity or resistance to [¹⁷⁷Lu]Lu-PSMA therapy. Clinical studies have linked resistance to mutations in growth factor genes (e.g. *EGFR*,* AR*) [61, 62], DNA damage repair genes (*ATM*,* BRCA1/2*) [61, 63, 64], and pathways such as *TP53* and *PI3K/AKT*. Alterations in these pathways, including *TP53* mutations [65–67], and *PTEN* loss or *PI3K/AKT* activation [45, 56, 68–70], are associated with poorer outcomes and radioresistance through mechanisms like enhanced DNA repair, apoptosis inhibition, and metabolic changes. Interestingly, in our analysis of five PSMA-high PDX models, the two good responders had genomic aberrations in *RB1*, while the partial responder and non-responders had *TP53* mutations, which may have impacted treatment response; however, interpretations are limited by the small sample size. Neither overall genomic alteration burden nor specific DDR gene mutations were associated with response in this cohort, consistent with emerging clinical data questioning the predictive value of DNA damage repair gene mutations in [^177^Lu]Lu-PSMA therapy [18, 19].

Plasma cell-free DNA (cfDNA), including its tumor-derived portion (circulating tumor DNA; ctDNA) provides a minimally-invasive means to assess molecular features associated with response to [¹⁷⁷Lu]Lu-PSMA therapy, with recent studies identifying candidate prognostic and predictive biomarkers, such as baseline ctDNA fraction and specific genomic alterations [58, 69–71]. Notably, acquired resistance has rarely been linked to new genomic events [71], highlighting the likely importance of transcriptional and epigenomic regulation in determining radiosensitivity. There are several emerging approaches that enable inference of gene expression from plasma cfDNA, including nucleosome profiling, fragmentomics and cell-free plasma chromatin immunoprecipitation sequencing [72]. To date, ctDNA methylation profiling demonstrates a strong ability to detect neuroendocrine prostate cancer, a phenotype that is generally refractory to [^177^Lu]Lu-PSMA therapy [73, 74]. Beyond phenotype, chromatin accessibility and transcriptional activity in key DNA repair and oncogene pathways may influence vulnerability to [^177^Lu]Lu-PSMA therapy. Integration of these clinical molecular features with functional studies in PDX models provides an opportunity to link genomics, epigenomic state, radiation response, and tumor growth dynamics.

Although genomic studies on [^177^Lu]Lu-PSMA clinical samples are increasing, transcriptomic analyses remain scarce. While some studies have examined transcriptomics in circulating tumor cells in the context of AR signalling and response to AR-directed therapies [75, 76] or other radiation types [77, 78], limited studies have examined prostate tumor transcriptomics in response to [^177^Lu]Lu-PSMA therapy [79]. However, that study focused primarily on immune markers (e.g. PDL-1/PDL-2), and no studies have yet linked broader transcriptomic profiles to [^177^Lu]Lu-PSMA therapy response.

Here, we assessed transcriptomic changes following [^177^Lu]Lu-PSMA treatment and found that the number of DEGs varied widely across tumors. Tumors that responded well generally exhibited a greater number of DEGs, including downregulation of genes involved in the cell cycle pathways and upregulation of apoptotic pathways, consistent with increased cell death. We also observed reduced expression of genes across multiple metabolic pathways, suggesting a coordinated suppression of metabolic activity in response to treatment. In addition, there was also evidence for a shift in the tumor microenvironment following [^177^Lu]Lu-PSMA treatment, with changes in hypoxia, angiogenesis and epithelial-mesenchymal transition pathways. This was combined with an upregulation of inflammatory pathways, including the interferon, TNFα and TGFβ signalling pathways, in the good responders that was not seen in the partial responders. While there was evidence for transcriptional changes in these inflammatory pathways based on RNA sequencing of the tumor cells, there was no change in immune cell infiltration into the tumors following treatment, as demonstrated by flow cytometry to assess tumor composition. However, this may be due to the limited immune cell repertoire in immunodeficient NSG, and further evaluation of the anti-tumor immune response to [^177^Lu]Lu-PSMA is required in an immunocompetent setting, for instance a syngeneic mouse model.

Baseline transcriptomic profiles may also contribute to tumor responses to [^177^Lu]Lu-PSMA. The PDX with the strongest response (PDX-27.1A-Cx), marked by sustained tumor regression and high radioligand uptake, exhibited baseline features suggestive of radiosensitivity, including low GSEA scores for hypoxia and ROS-related pathways. In well-oxygenated tumors like PDX-27.1A-Cx, radiation-induced free radicals are more likely to accumulate and cause DNA damage. Conversely, hypoxic tumors, such as PDX-463.8A, may produce fewer ROS, limiting DNA damage and promoting resistance. Hypoxia also contributes to radioresistance through increased antioxidant activity and metabolic reprogramming [80, 81]. Thus, reduced hypoxia and antioxidant capacity at baseline may enhance radiosensitivity, while elevated activity in these pathways may underlie resistance in poor responders.

The multiple factors influencing radiosensitivity and resistance, at both a tissue and cellular level, reported here further supports the rationale for combination therapies to enhance tumor radiosensitivity and therapeutic efficacy. Several clinical trials are currently evaluating the benefit of combining [^177^Lu]Lu-PSMA therapy with radiosensitizing agents, including PARP inhibitors, androgen receptor pathway inhibitors, chemotherapy, and immune checkpoint blockade [82–86]. Targeting tumor hypoxia may be another avenue to promote radiosensitisation. Given the diversity and tumor-specific nature of resistance mechanisms, evident even in a small subset of PDX models, a key challenge will be identifying the most appropriate combination therapy for individual patients. Further assessment of baseline features, including transcriptomic signatures identified in this study as well as other prognostic markers, such as ctDNA fraction or DNA damage response markers in peripheral blood lymphocytes [71, 87], may enable a multimodal approach to improve patient stratification. An additional consideration will be the potential toxicity of combination treatment strategies, with reports of increased tissue radiosensitivity following radiosensitizing agents [88].

An unexpected finding was that PDX-387.38A-Cx, classified as a non-responder based on tumor growth, exhibited substantial changes in cellular composition and transcriptomic profile following [¹⁷⁷Lu]Lu-PSMA treatment. This tumor displays a rare amphicrine phenotype, co-expressing both AR and NE markers, and was derived from a patient that had been treated with [^177^Lu]Lu-PSMA prior to tissue collection. Whilst the patient initially responded to [^177^Lu]Lu-PSMA, disease progression occurred and the patient unfortunately died shortly after treatment when the autopsy sample was collected; however, we do not know whether the specific lesion sampled for PDX establishment responded to treatment or not. The high number of DEGs observed following treatment, along with the downregulation of cell cycle pathways and observed histological changes, suggests that this PDX is biologically responsive to radioligand therapy despite showing no change in overall tumor volume. The disconnect between observable growth changes, reduction in tumor cell proportion, and transcriptomic modifications may be due to a high presence of necrotic cells, resulting in maintenance of tumor mass. Alternatively, there could be a delay in the onset of tumor reduction following treatment, as was observed with the delayed response to long-term treatment with PDX-463.8A. The histological and molecular changes could also suggest potential modulation of the tumor microenvironment, possibly priming it for future therapies, including rechallenge with [¹⁷⁷Lu]Lu-PSMA alone or in combination. Further investigation with longer term experimental time points are needed to explore these possibilities.

## Conclusions

Our study shows that individual PDX tumors exhibit diverse PSMA expression and receptor density, genomic features and baseline transcriptional landscapes, highlighting the complexity of [^177^Lu]Lu-PSMA treatment response. Integrating baseline transcriptomic analysis with DNA damage response, PSMA expression and genomic profiles may help identify features linked to sensitivity or resistance to [¹⁷⁷Lu]Lu-PSMA therapy. Importantly, PDX models offer a valuable preclinical platform to further explore these mechanisms and test combination therapies, thereby advancing prostate cancer research and treatment.

## Supplementary Information

Supplementary Material 1.

## Data Availability

The targeted DNA sequencing and bulk RNA-sequencing data that support the findings of this study have been deposited in the NCBI’s dbGaP ( https://www.ncbi.nlm.nih.gov/gap/ ) accession number phs003369.v3.p1. Source data are available as Source Data File. The remaining data are available within the Article, Supplementary Information or from the corresponding author upon request. To request access to MURAL PDXs and/or biospecimens, including organoids, researchers should contact Dr. Melissa Papargiris, MURAL Project Manager (melissa.papargiris@monash.edu) to initiate an Expression of Interest. Researchers would need to provide evidence of institutional approval to experiment with human PDX tumors and research would be conducted under the conditions of a Materials Transfer Agreement.

## Abbreviations

Adeno: Adenocarcinoma
ADT: Androgen deprivation therapy
AR: Androgen receptor
cCasp3: Cleaved-caspase 3
CR: Complete response
cfDNA: Cell-free DNA
ctDNA: Circulating tumor DNA
DEG: Differentially expressed gene
DSB: Double strand break
EMT: Epithelial-mesenchymal transition
GSEA: Gene set enrichment analysis
HIF1α: Hypoxia-inducible factor 1α
IHC: Immunohistochemistry
MURAL: Melbourne Urological Research Alliance
NE: Neuroendocrine
NES: Normalized enrichment score
PARPi: Poly (ADP-ribose) polymerase inhibitor
PCA: Principal component analysis
PD: Progressive disease
PDX: Patient-derived xenograft
PET: Positron emission tomography
PGA: Percent genome alteration
PR: Partial response
PSMA: Prostate-specific membrane antigen
ORC: Objective response criteria
ROS: Reactive oxygen species
SAHF: Senescence-associated heterochromatin foci
SASP: Senescence-associated secretory phenotype
SD: Stable disease
SEM: Standard error of the mean
SUV: Standardized uptake value
