# Supplementary material for: Variable cellular and radiobiological effects of [177Lu]Lu-PSMA-I&T in patient-derived models of prostate cancer
Source: J Exp Clin Cancer Res. 2026 Feb 13;45:76. doi: 10.1186/s13046-026-03659-w (PMC13020377; doi:10.1186/s13046-026-03659-w)
Supplement: Supplementary file 1 — Supplementary Material 1. [file 13046_2026_3659_MOESM1_ESM.docx]

**SUPPLEMENTARY MATERIAL**
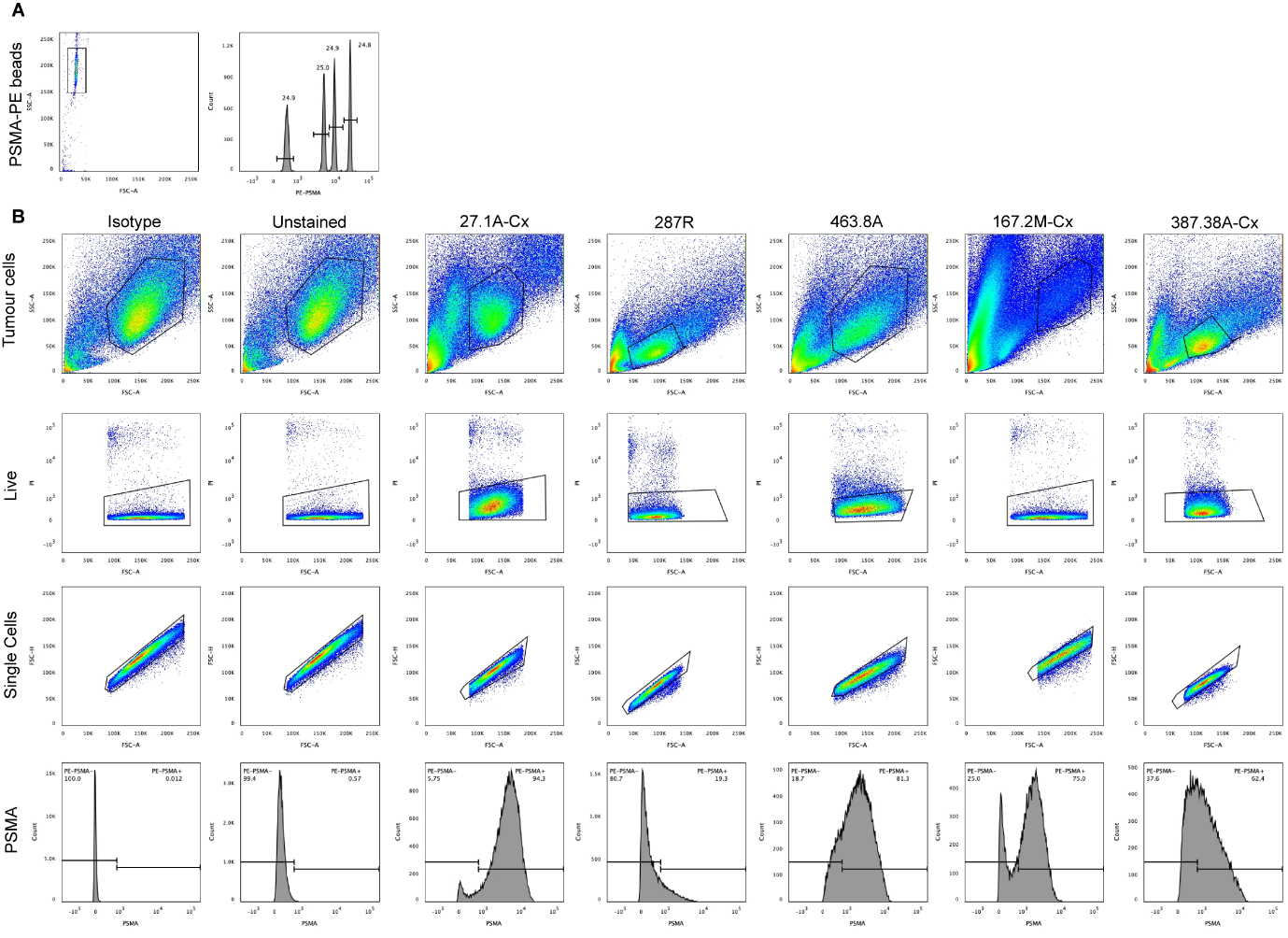


**Supplementary Figure 1: Gating strategy for PSMA receptor density cytometric bead assay for five PSMA-high PDXs. A)** Gating of PE beads for quantitation of receptor density. **B)** Gating for PDX dependant on cell size: tumor cells, live cells, and single cells were gated, and PSMA positivity was gated based on isotype and unstained controls.

**
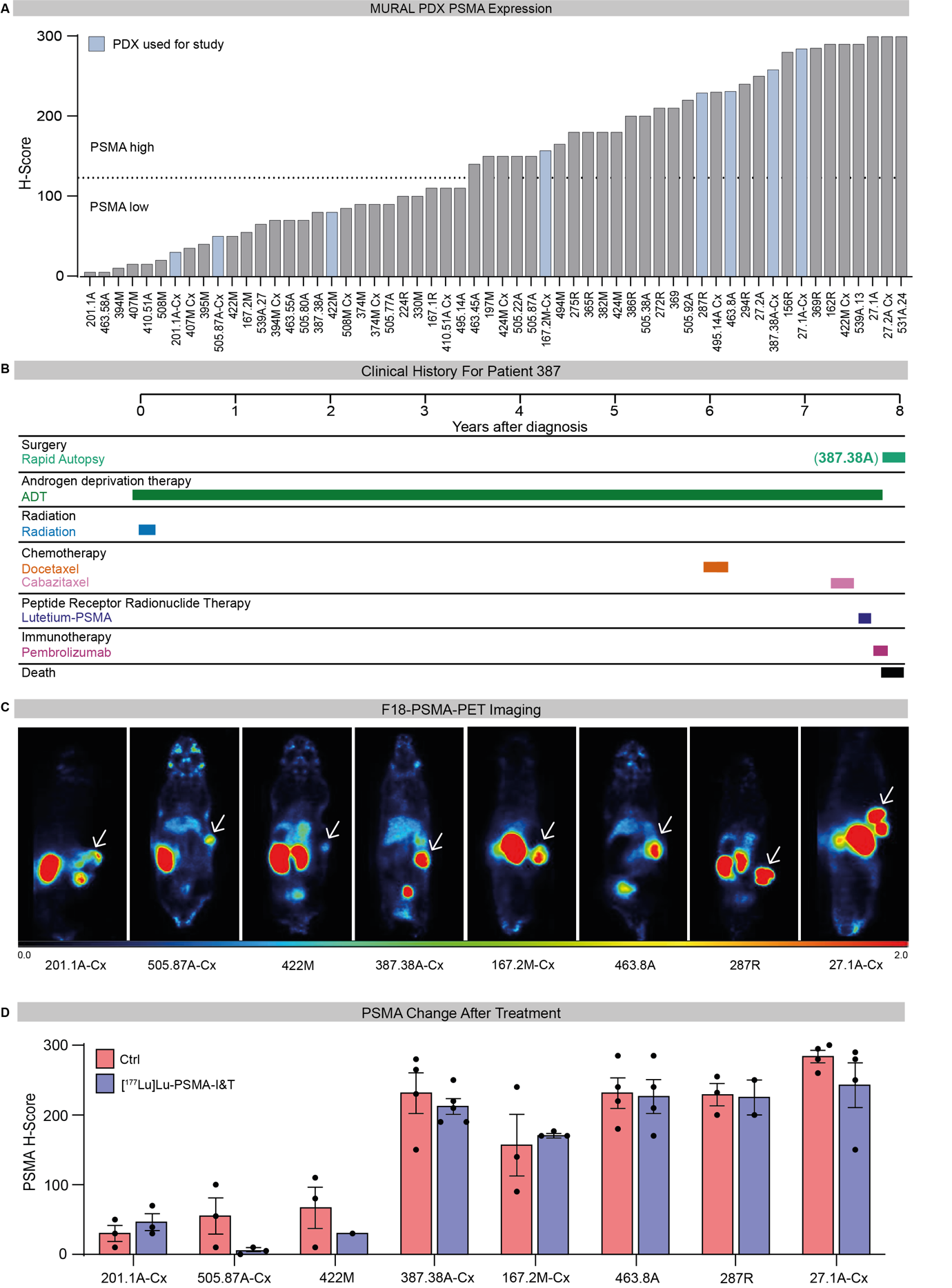
**

**Supplementary Figure 2: PSMA expression in prostate cancer PDXs. A)** PSMA expression by H-score, determined by immunohistochemical staining, in the MURAL collection of prostate cancer PDXs. Only PSMA-positive tumors (H score >10) are shown. Tumors highlighted in blue were treated with [^177^Lu]Lu-PSMA. **B**) Treatment timeline for Patient 387 showing each treatment the patient received from diagnosis to death. The sample used to establish PDX-387.38 is shown in brackets. **C)** Representative F18-PSMA-PET images of eight PDXs with white arrows indicating tumors. **D)** PSMA expression in control and treated tumors assessed by immunohistochemical staining at 2 weeks post a single 60 MBq injection of [^177^Lu]Lu-PSMA (n=2-5 tumors per PDX). Unpaired students t-test between control and treated tumors of each PDX showed no significant differences in PSMA expression.


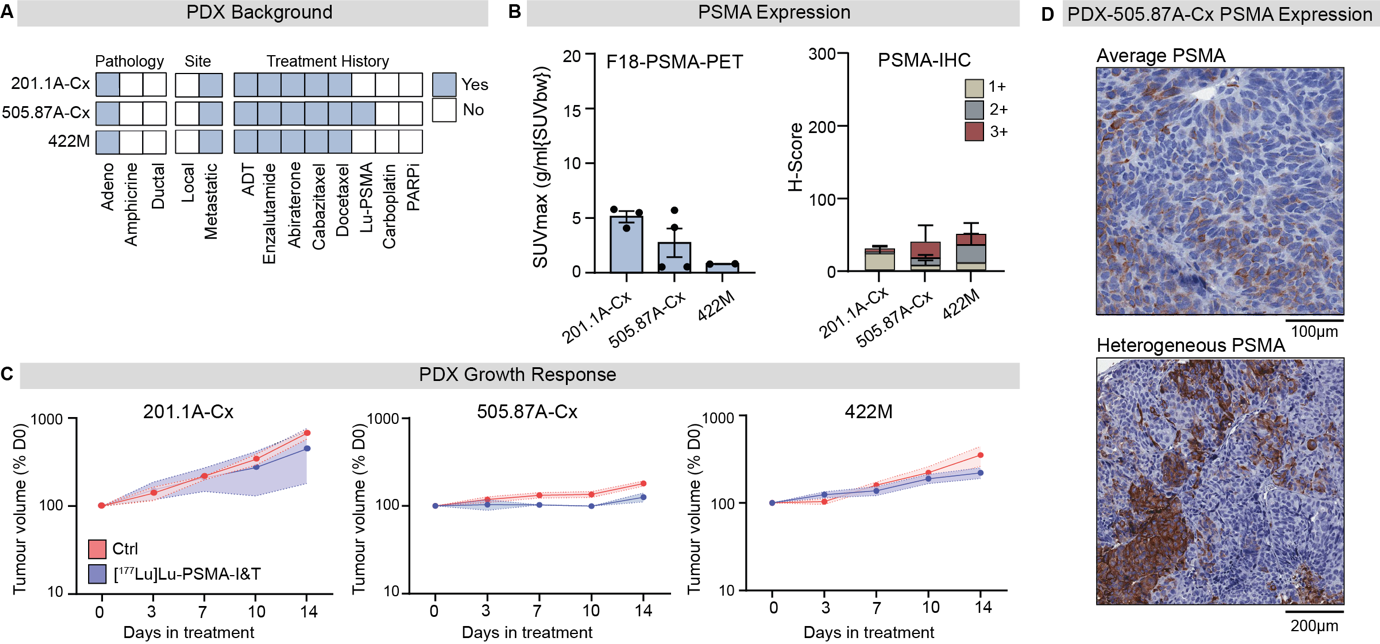


**Supplementary Figure 3: PDX background, PSMA expression, and growth response to [^177^Lu]Lu-PSMA administration in three PSMA-low PDXs. A)** Pathology, site of origin and treatment history of tumors that were obtained to generate PSMA-low PDXs. **B)** PSMA expression by F18-PET and immunohistochemical staining in PSMA-low tumors. **C)** PSMA-low tumor volume (mean ± SEM) following intravenous injection of 60MBq (± 8MBq) [^177^Lu]Lu-PSMA-I&T or control (saline) on day 0. Sample sizes for individual PDX models: 201.1A-Cx n=3, 505.87A-Cx n=3/4, 422M n=3/4. **D)** PSMA intratumoral heterogeneity by immunohistochemistry in two representative tumors from PDX-505.87A-Cx, showing distinct regions of high PSMA expression next to PSMA-negative cells.


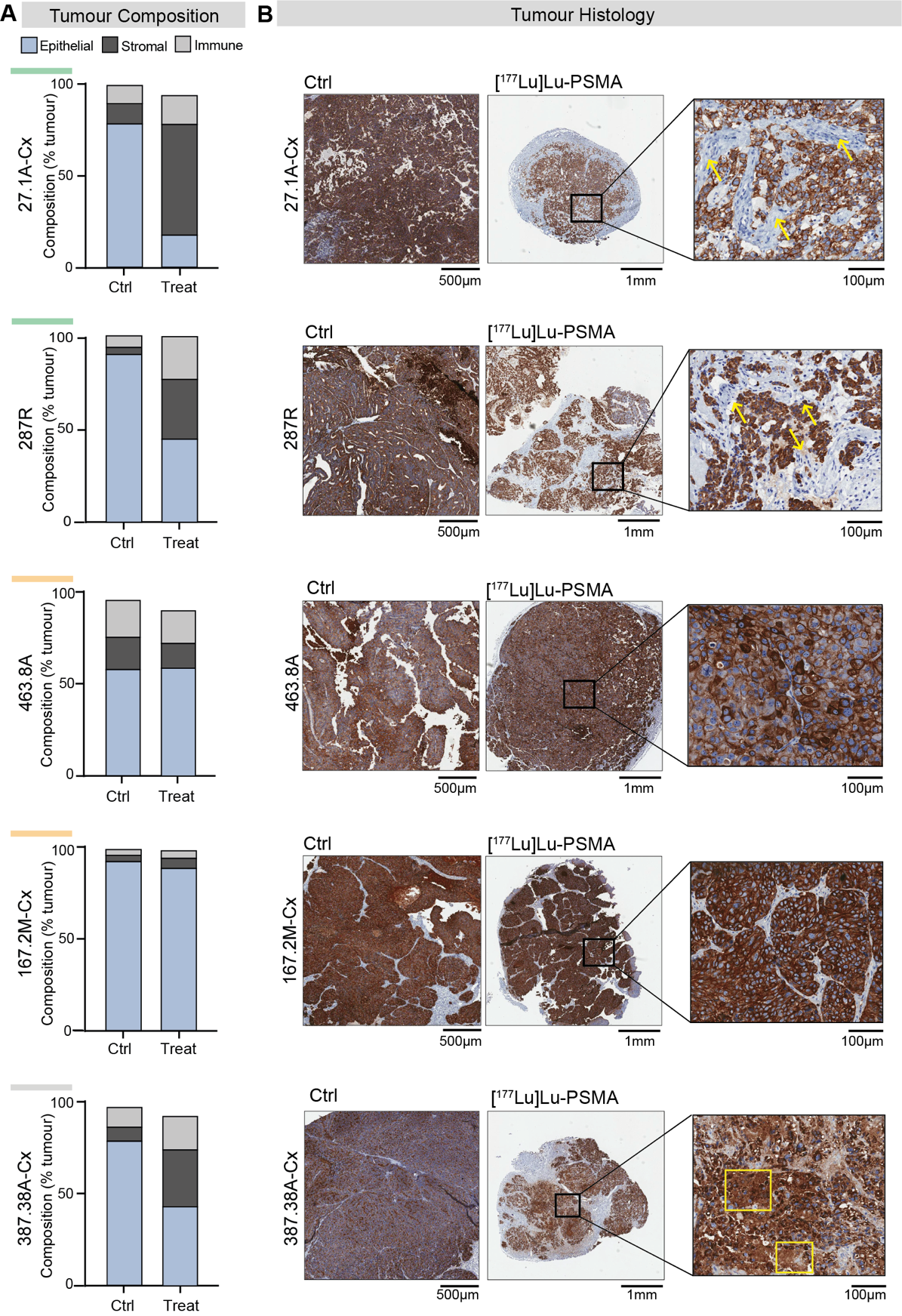


**Supplementary Figure 4: Compositional changes in tumors after [^177^Lu]Lu-PSMA treatment.**

**A**) Cellular composition of tumors. Data represents the mean of 4 tumors per group. Immune (mouse); CD45^+^/EpCam^-^. Tumor epithelium (human) EpCam^+^/CD45^-^. Stromal (mouse); CD45^-^/EpCam^-^. **B**) Representative tumor composition in control tumors and and tumors two weeks post-treatment. Immunohistochemical staining of human epithelial marker CK8/18; yellow arrows indicate stroma (mouse); yellow boxes indicate areas of necrosis.

**
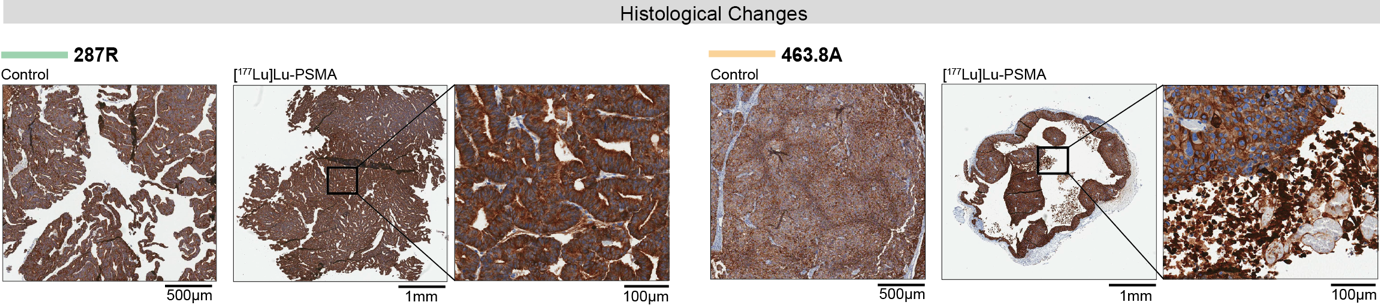
Supplementary Figure 5: Long-term histological changes in tumors after [^177^Lu]Lu-PSMA treatment.** Histological changes in tumors at the time of tissue collection after a maximum of 20 weeks following a single intravenous injection of 60 MBq (± 8MBq) [^177^Lu]Lu-PSMA-I&T or control (saline). Histological changes visualised by ck8/18 immunohistochemical staining. No change was observed in PDX-287R histopathology following [^177^Lu]Lu-PSMA administration, whereas large necrotic areas were observed in PDX-463.8A tumours following treatment compared to controls (insets).

**
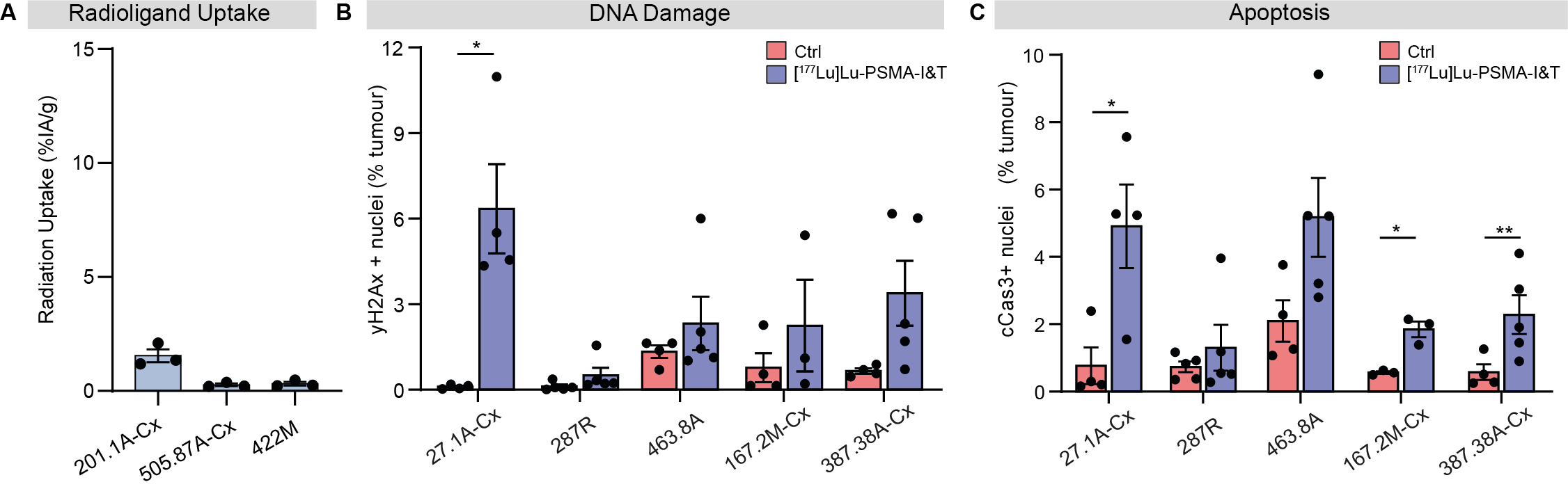
Supplementary Figure 6: DNA damage and cell death two weeks post-treatment with [^177^Lu]Lu-PSMA. A)** Whole tumor radioligand uptake in three PSMA-low PDXs, measured by gamma counter 24h after [^177^Lu]Lu-PSMA administration and expressed as the percent of injected activity per gram of tumor tissue (n = 3 tumors per PDX). **B)** Expression of DNA damage repair marker yH2Ax in control and treated samples from PSMA-high PDXs, analysed two weeks post-treatment with 60 MBq [^177^Lu]Lu-PSMA-I&T (n = 3-5 tumors per group per PDX). Immunohistochemical staining was quantified as total percentage of 2+ plus 3+ positive nuclei in the tumor. **C)** Intrinsic apoptotic protein cleaved-caspase3 (cCas3) in control and treated samples from PSMA-high PDXs two-weeks post-treatment with 60 MBq [^177^Lu]Lu-PSMA-I&T by immunohistochemical staining and quantitated as total percentage of 2+ and 3+ nuclei in the tumor (n = 3-5 tumors per group per PDX). Unpaired students t-test between control and treated groups (*p<0.05 **p<0.01).

**
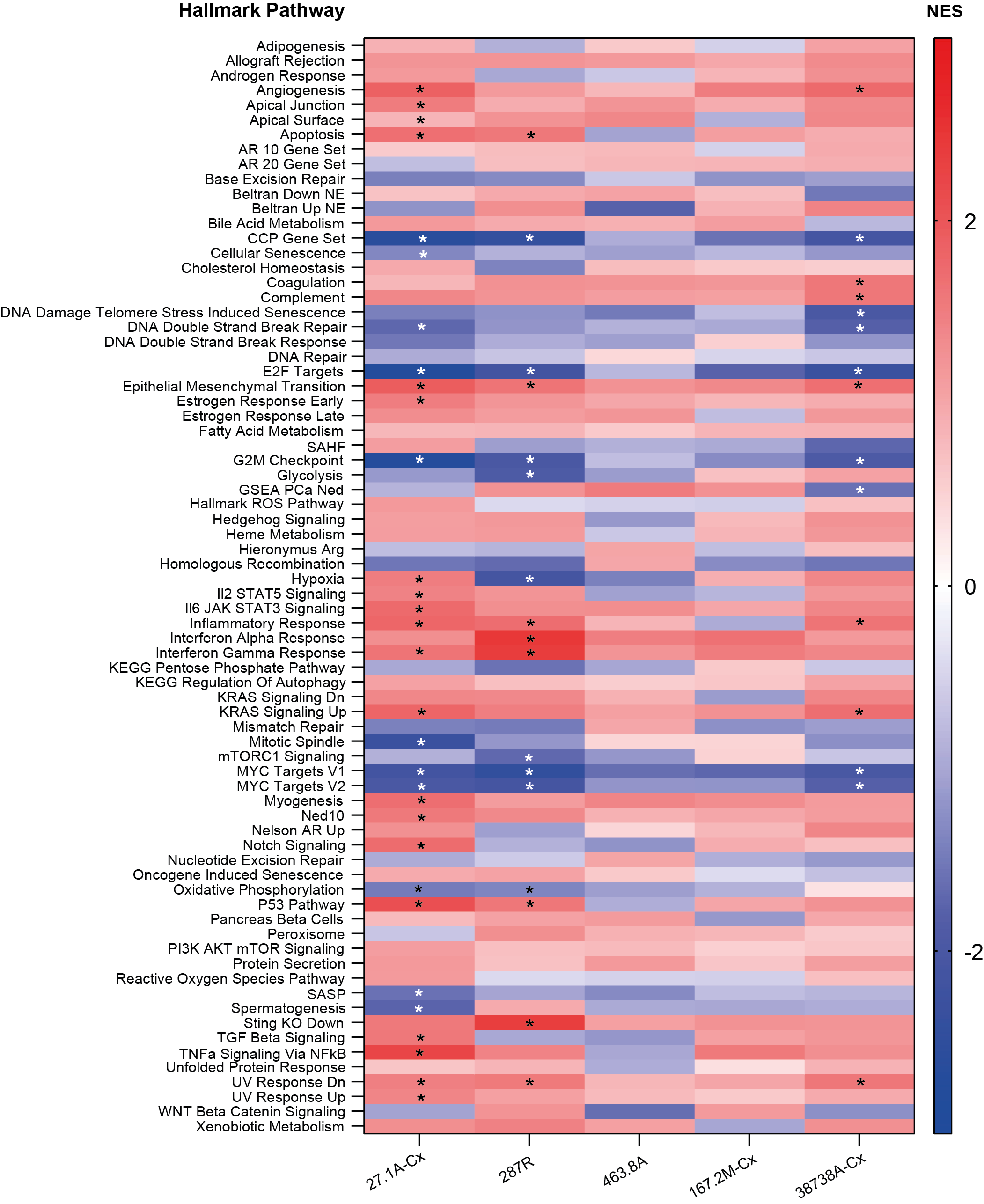
Supplementary Figure 7: Transcriptomic regulation of hallmark cancer pathways** **two weeks post-treatment with [^177^Lu]Lu-PSMA.** Normalised Enrichment Scores (NES) for all hallmark cancer pathways analysed in five PSMA-high PDXs. Samples were acquired two weeks after [^177^Lu]Lu-PSMA treatment and compared to controls at this timepoint. Asterisks denote significantly enriched pathways (padj<0.05).

**Supplementary Table 1: Antibody conditions for immunohistochemistry and flow cytometry**
